# Harmonizing Innovations: An In-Depth Comparative Review on the Formulation, Applications, and Future Perspectives of Aerogels and Hydrogels in Pharmaceutical Sciences

**DOI:** 10.3390/gels10100663

**Published:** 2024-10-17

**Authors:** Nour Alhuda Alaghawani, Hala Alkhatib, Layla Elmancy, Anis Daou

**Affiliations:** Pharmaceutical Sciences Department, College of Pharmacy, QU Health, Qatar University, Doha P.O. Box 2713, Qatar; na1901676@student.qu.edu.qa (N.A.A.); ha1802569@student.qu.edu.qa (H.A.); le1901995@student.qu.edu.qa (L.E.)

**Keywords:** gels, hydrogels, aerogels, formulation, antimicrobial applications, sol–gel chemistry, drug loading, comparative analysis

## Abstract

Gels, specifically hydrogels and aerogels, have emerged as versatile materials with profound implications in pharmaceutical sciences. This comprehensive review looks into detail at hydrogels and aerogels, providing a general introduction to gels as a foundation. The paper is then divided into distinct sections for hydrogels and aerogels, each delving into their unique formulations, advantages, disadvantages, and applications. In the realm of hydrogels, we scrutinize the intricacies of formulation, highlighting the versatile advantages they offer. Conversely, potential limitations are explored, paving the way for a detailed discussion on their applications, with a specific focus on their role in antimicrobial applications. Shifting focus to aerogels, a thorough overview is presented, followed by a detailed explanation of the complex formulation process involving sol–gel chemistry; aging; solvent exchange; and drying techniques, including freeze drying, supercritical drying, and ambient-pressure drying (APD). The intricacies of drug loading and release from aerogels are addressed, providing insights into their pharmaceutical potential. The advantages and disadvantages of aerogels are examined, accompanied by an exploration of their applications, with a specific emphasis on antimicrobial uses. The review culminates in a comparative analysis, juxtaposing the advantages and disadvantages of hydrogels and aerogels. Furthermore, the current research and development trends in the applications of these gels in pharmaceutical sciences are discussed, providing a holistic view of their potential and impact. This review serves as a comprehensive guide for researchers, practitioners, and enthusiasts, seeking a deeper understanding of the distinctive attributes and applications of hydrogels and aerogels in the ever-evolving research concerning pharmaceutical sciences.

## 1. Introduction

Gels are semisolid formulations used for the topical application of drugs on the skin or mucoidal membrane [1]. The USP defines gels as semisolid systems containing either suspensions of small inorganic particles, or large organic molecules interpenetrated by a liquid [2]. Gels consist of two interoperating systems, where the colloidal particles are evenly distributed throughout the dispersion medium, which is a solvent forming a matrix [1]. The gels are formulated by adding a gelling agent, which may be a synthetic/semi-synthetic polymer or a natural polymer, into an aqueous or inorganic solvent [3,4,5]. Besides the polymer and the solvent, gels are composed of several ingredients, such as stabilizers, dispersing agents, penetration enhancers, and preservatives. Additionally, their structure allows for multiple advantages, including structural strength and increased adherence to the skin’s surface, aiding in longer retention [6]. Some of the major advantages of gel formulations over other semisolid dosage forms (such as creams and ointments) are as follows: Firstly, they are simpler to formulate, having fewer steps in comparison to creams and ointments. Furthermore, gels are not sticky or greasy, thus making them easy to apply. Additionally, gels exhibit excellent spreadability and can provide a cooling effect as their solvent evaporates. Finally, they can harbor both polar and nonpolar drugs [4,6,7]. Based on the nature of the dispersed phase, as well as the network structure, gels can be classified into either hydrogels or aerogels. Hydrogels are hydrophilic, three-dimensional networks which are capable of absorbing and retaining significant amounts of water or biological fluids within their structure, as it resembles biological tissues due to its large water content and soft consistency [8]. The presence of hydrophilic functional groups attached to their polymeric backbone allows hydrogels to absorb fluids while resisting dissolution, due to the chemical or physical cross-links between network chains [9]. During the last two decades, synthetic hydrogels were gradually replacing natural hydrogels due to their high capacity of absorption, high gel strength, and long service life, in addition to their stability when exposed to conditions of great fluctuations in temperature and humidity [9]. Hydrogels can be synthesized in many different chemical processes, including one-step procedures like polymerization and parallel cross-linking of multifunctional monomers, as well as multistep procedures that involve the synthesis of polymer molecules with reactive groups and their subsequent cross-linking, where they mainly react with polymers that include suitable cross-linking agents [9]. The products of hydrogels can be classified based on many different aspects, including the source (natural or synthetic origin), polymeric composition (homo-polymeric hydrogels, co-polymeric hydrogels, or multi-polymer interpenetrating polymeric hydrogel), configuration (amorphous, semi-crystalline, or crystalline), type of cross-linking (chemical or physical cross-links), or the network electrical charge (neutral, ionic, ampholytic, or zwitterionic) [9]. Hydrogels are one of the upcoming classes of polymer-based systems that hold many biomedical and pharmaceutical applications [10]. One of their important applications is their use as drug-delivery systems for efficient therapy, as they are excellent candidates for controlled release devices, bio-adhesives, or targetable devices of therapeutic agents, mainly due to their soft, tissue-like consistency and their high biocompatibility [11]. Using hydrogels as delivery devices can be useful in oral, rectal, ocular, epidermal, and subcutaneous applications [8].

In the last decade, aerogels have witnessed an increased interest in pharmaceutical and biomedical applications as a result of their exceptional properties and versatility. Much like hydrogels, the structure of aerogels comprises organic, synthetic, or colloidal material arranged in a solid porous three-dimensional network [12]. The main differential feature in their structure, however, is the gaseous phase found in the pores of the aerogel network compared to the liquid phase found in the network of their hydrogel counterparts. This can further be deduced by looking at the IUPAC definition of aerogels in which they are described as microporous solid gels with a gaseous dispersed phase [13]. As a result of this unique phase, aerogels are often described in the literature as being the lightest solids on earth, where they possess a high number of mesopores (2–50 nm wide) that contribute to the massive empty volume fraction in their network and allows them to have an exceptionally low density of around 0.0001 to 0.2 g/cm^3^ [12]. In addition to that, the numerous pores with adjustable sizes that are found in the aerogels’ structure allow for these gels to have a large surface area, making them one of a kind for drug loading and delivery [14,15].

In general, during the formulation of aerogels three main steps are typically followed: cross-linking (sol–gel formation), aging, and finally drying [14], with the latter being a crucial step in removing any fluids, to prevent the development of surface tension and the collapse of the aerogel pores [12,14,16]; this, in turn, maintains the high surface area and unique low density of the nanoporous three-dimensional morphology of these gels. Aerogels can be found grouped into two main subcategories, which are organic aerogels: those made of biopolymers, such as cellulose and chitin; and inorganic/synthetic aerogels, which are those composed of silica, alumina, and other metal oxides. Recently, biopolymer aerogels have undergone an interesting attraction in pharmaceutical sciences as a result of their high surface area and porous structure, which allowed for them to have an increased bioavailability, an application in drug transportation, drug loading, and sterile wound dressing [17]. Other relevant applications of aerogels include theranostics, transdermal drug delivery, nasal drug delivery, and pulmonary drug administration [12].

## 2. Hydrogels

Hydrogels are one form of gels composed of cross-linked hydrophilic polymeric networks which are capable of absorbing and retaining significant amounts of water or biological fluids [10]. The hydrophilic functional groups attached to the polymeric backbone become hydrated in aqueous media, thus forming a hydrogel structure, while the cross-links between the network chains are present to prevent the dissolution of the polymer chains before use [18]. Both the three-dimensional structure of the hydrogels and their thermodynamic compatibility with water allow them to swell and hold water within their matrix [18].

In the past 50 years, hydrogels have received a considerable amount of attention due to their exceptional promise in a wide range of applications, and in particular, the medical and pharmaceutical sectors [9]. Hydrogels resemble natural living tissues more than any other class of synthetic biomaterials since they possess a high degree of flexibility which is very similar to that of natural tissue, in addition to their large water content and soft consistency [9]. The high-water content, swelling characteristics, and diffusional behavior of hydrogels contributes to their biocompatibility, biodegradability, porosity, and mechanical strength, making them a potential candidate in tissue engineering, in the biomedical field, and in pharmaceutical applications [8]. Besides this, hydrogels play an important role in the development of drug-delivery systems. Hence, hydrogels can be used as linings for artificial hearts, lubricants, contact lenses, materials for artificial skin, and for the delivery of various therapeutic agents [8].

### 2.1. Hydrogel Formulation

**Figure 1:**
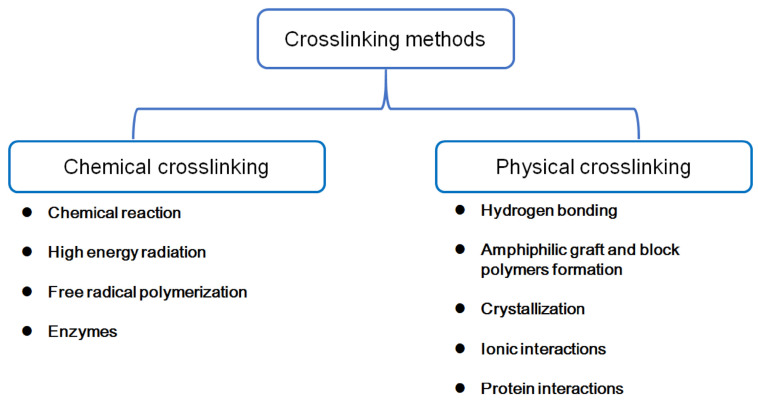
Methods of chemical and physical cross-linking for hydrogels preparation [23].

Hydrogels are formulated using a variety of methods and techniques depending on the specific requirements of the intended pharmaceutical application. The formulation process involves selecting a suitable polymer, cross-linking agents, and additives, as well as determining the appropriate processing conditions. The first critical step in formulating hydrogels is choosing the appropriate polymer. Hydrogels can be prepared from either synthetic polymers or natural polymers, as shown in Table 1 [19]. The most preferred polymers are the hydrophilic polymers (like polyacrylic acid, polyvinyl alcohol, and polyethylene glycol) that can absorb and retain water while maintaining their structural integrity [20]. The next step in hydrogel preparation is the process of cross-linking, which involves linking the polymer chains to form a stable 3D network [18]. Cross-linking stabilizes the mutual position of polymeric chains to prevent the hydrogel from dissolving before use [21]. There are many different cross-linking methods used in designing hydrogels, and these methods fall into two main classifications: the physical and chemical cross-linking methods, as shown in Figure 1 [21,22,23].

Physically cross-linked hydrogels can be synthesized by protein interaction, ionic interaction, hydrogen bonding, stereo-complex formation, crystallization, and hydrophobized polysaccharides [21]. Hence, in physical hydrogels, the bonding between the polymeric chains is due to non-covalent (secondary forces) bonding, such as van der Waals interactions, hydrogen bonding, ionic forces, stereo-complexation, and hydrophobic forces [20]. These physical interactions present between the polymer chains are not very strong; hence, physical hydrogels are fragile, disordered, and mechanically weak when exposed to environmental changes, since they show a reversible (sol–gel transition) response to external stimuli, like changes in temperature and pH [20].

**Figure 2:**
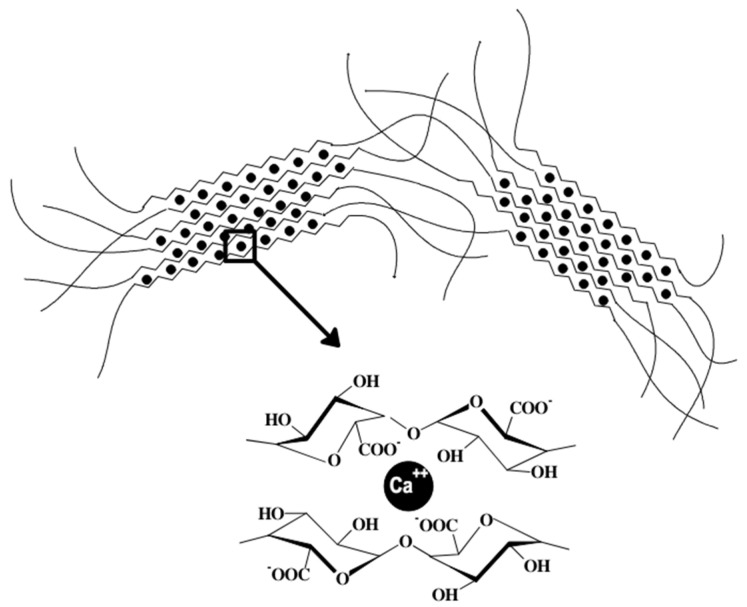
Gelation of sodium alginate by addition of calcium ions [24].

- In ionic cross-linking, hydrogels are formed by utilizing the electrostatic interactions between positively and negatively charged polymers [21]. Alginate may be cross-linked via divalent cations such as calcium ions, which cross-link the alginate chains through ionic interactions, as shown in Figure 2 [24]. Cross-linking is performed at physiological pH and at room temperature [18].
  ▪ Ionic cross-linking reaction:
  ▪ $Alginate-Na+{Ca}^{2+}⟶Alginate-Ca$
- For stereo-complex formation, a hydrogel is formed through the cross-linking between lactic acid oligomers of opposite chirality [21].
- Various polysaccharides, like chitosan, dextran, pullulan, and carboxymethyl curdlan, are used for the preparation of physically cross-linked hydrogels through hydrophobic modification [21]. Hence, the hydrogel is formed as a result of the polymer swelling and absorbing water due to hydrophobic interactions [21].

Chemically cross-linked hydrogels (permanent hydrogels) involve covalent bonding between the polymer chains [20]. Chemical hydrogels are synthesized by chain-growth polymerization, gamma and electron beam polymerization, and addition and condensation polymerization [21]. These various chemical cross-linking methods utilize small molecules (like formaldehyde, genipin, glutaraldehyde, and diglycidyl ether) to form covalent bonds with the polymers, thus resulting in the stabilization of the network through condensation reaction or free radical mechanisms [21]. Unlike physical hydrogels, chemical hydrogels do not dissolve in the surrounding medium, and, thus, they do not show a reversible response in response to environmental changes, due to the presence of strong covalent bonding between the polymeric chains [20].

- Chain-growth polymerization includes free radical polymerization, controlled free radical polymerization, and anionic and cationic polymerization [21], performed through three processes, initiation, propagation, and termination [21]. Through initiation, a free radical will activate monomers, and those monomers will form polymers in a chain-link-like fashion [25].
  ▪ Acrylate-based hydrogels are formed by free radical polymerization. Where acrylic acid or acrylamide monomers are polymerized using a free radical initiator (e.g., ammonium persulfate) in the presence of a cross-linker (e.g., N,N’-methylenebisacrylamide).
  ▪ $nAcrylic\;Acid+Cross-linker\;\overset{Initiator}{\to }\;\left(Polyacrylic\;Acid\right)n$
- Gamma and electron beam polymerization involves high-energy electromagnetic irradiation as a cross-linker. This potent radiation effectively cross-links water-soluble monomer or polymer ends, without the need for adding a cross-linking agent [21]. During irradiation, using a gamma or electron beam, aqueous solutions of monomers are polymerized to form a hydrogel [21].
- Stepwise polymerization involves the use of polycondensation reactions to allow functional groups of monomers to react and join each other through covalent bonds [25].

**Table 1:** Different polymers used in developing hydrogels.

	Natural Polymers	Synthetic Polymers
Description	Derived from natural sources, such as plants, animals, or microorganisms. Biocompatible, biodegradable, and exhibit inherent bioactivity.	Chemically synthesized in the laboratory. Derived from monomers through polymerization reactions.
Examples	Collagen [26]	Poly(acrylic acid) (PAA) [19]
	Alginate	Poly(N-isopropylacrylamide) (PNIPAAm) [19]
	Chitosan [27]	Poly(ethylene glycol) (PEG) [19]
	Hyaluronic acid	Poly(vinyl alcohol) (PVA) [19]
	Gelatin	Poly(HEMA) (hydroxyethyl methacrylate) [19]
	Dextran	Poly-lactic acid (PLA) [28]
	Fibrin	Polyglycolic acid
	Pectin	Polyiminocarbonates
	Carrageenan	Polyethylene glycol diacrylate/dimethacrylate
	Carboxymethyl chitin	Polyvinyl pyrrolidone (PVP) [29]
	Guar gum	Polyethylene imine
	Cellulose	Polymethacrylate
	Xanthan gum	Polyvinyl acetate
	Chitin	Polymethyl methacrylate
	Lignin	Polycaprolactone [30]
	Starch	Poly(ethylene oxide) (PEO)
	Carrageenan	Poly(2-hydroxyethyl methacrylate) (PHEMA)

**Natural and Synthetic Polymers used in the Formulation of Hydrogels:**

Hydrogels can be categorized as natural, synthetic, or a combination of both. Hydrogels obtained from naturally occurring polymers are called natural polymer hydrogels. Natural polymers can be obtained from diverse natural sources like animals, plants, or microorganisms. Natural polymers can be classified into various classes depending on their chemical structure: [26]

- Polysaccharides (chitin, chitosan, cellulose, starch, gums, alginate, and carrageenan);
- Biological polymers (nucleic acid and DNA);
- Polyamides (collagen);
- Polyphenols (lignin);
- Organic polyesters;
- Inorganic polyesters (polyphosphazene);
- Polyanhydrides (poly sebacic acid).

These polymers have well-defined, larger structures formed by covalently bonded monomeric units [20]. Natural polymers are abundant, non-toxic, inexpensive, easy to access, biodegradable, and biocompatible; exhibit inherent bioactivity; and possess other attractive biological properties [20], making them suitable for a wide range of biomedical applications, such as controlled and targeted release of drugs, and biomedical engineering [19]. Hydrogels formed from naturally occurring polymers, especially polysaccharides and proteins, are similar to our cells extracellular matrix due to their natural origin, making them easily identifiable by the body’s cells; thus, they possess the capabilities to support cell growth, tissue regeneration, and wound healing [19,31]. They are also able to provide a favorable microenvironment for encapsulated cells or therapeutic agents [32]. Moreover, due to their inherent bioactivity, natural polymers allow for bioactive molecule incorporation or modification to enhance specific functionalities [19].

Synthetic polymer-based hydrogels are hydrogels composed of polymers that are chemically synthesized in the laboratory [20]. They contain synthetic polymers that are typically derived from monomers through polymerization reactions, allowing for precise control over their physical and chemical structures, as well as properties and functionality compared to natural hydrogels. Thus, synthetic polymers offer more flexibility to tune the mechanical properties of the hydrogels, their reproducibility, and their ability to incorporate various functionalities for specific applications [20,33]. Synthetic polymers can be produced with long-chain structures and high molecular weight; however, synthetic polymer hydrogels possess a lower biological activity than natural hydrogels. These hydrogels can be synthesized via numerous ways, employing polymerizable vinyl monomers or chemical cross-linking of polymers. The most commonly used synthetic polymers are polycaprolactone, poly(vinyl pyrrolidone) (PVP), poly (lactic acid) (PLA), poly(ethylene glycol) (PEG), and poly(vinyl alcohol) (PVA) [20]. Synthetic hydrogels offer advantages such as precise control over their chemical and physical properties, mechanical strength, and stability [34]. They can be engineered to exhibit specific characteristics, such as controlled drug release, stimuli responsiveness, and biodegradability [35,36]. Furthermore, synthetic polymers can be modified through various chemical reactions, allowing for the incorporation of bioactive molecules, peptides, or targeting ligands to enhance their functionality and specificity [37]. Synthetic polymer-based hydrogels find applications in various fields, including drug-delivery systems, tissue engineering, biosensors, and wound healing [38].

**In situ gelling hydrogels**

Recently, an increasing number of in situ forming systems have been reported in the literature for various biomedical applications of hydrogels, including drug delivery, cell encapsulation, and tissue repair [39]. An in situ gelling system is a formulation that is in the form of a solution or an injectable fluid before it is introduced into the body in a minimally invasive manner, but it transforms into a gel under a variety of physiological conditions, within the desired tissue, organ, or body cavity. The basic advantage is the ability of the in situ hydrogel to change its properties, such as mechanical properties, swelling capacity, hydrophilicity, or permeability of bioactive molecules (sol-to-gel transition) under the effect of various stimuli, including changes in temperature, pH, and ionic strength; exposure to electromagnetic radiation; and the presence of specific molecules [39]. Stimuli-responsive properties can enable the formulation of novel targeted drugs and control drug release through non-intravenous administration. They can also delay the effect of opsonization via low blood contact.

Injectable gel-forming matrices offer several advantages over systems shaped into their final form before implantation. For example, injectable materials do not require a surgical procedure for placement, and various therapeutic agents can be incorporated by simple mixing. When they are used to fill in a cavity or a defect, their flowing nature enables a good fit. In situ implant formation can occur as a result of either a physical or chemical change in the system [39].

There are many different types of in situ gelling systems, such as temperature-sensitive systems, thiolated hydrogels, pH-sensitive systems, ion-sensitive systems, and multi-responsive systems, as shown in Table 2 [40].

**Figure 3:**
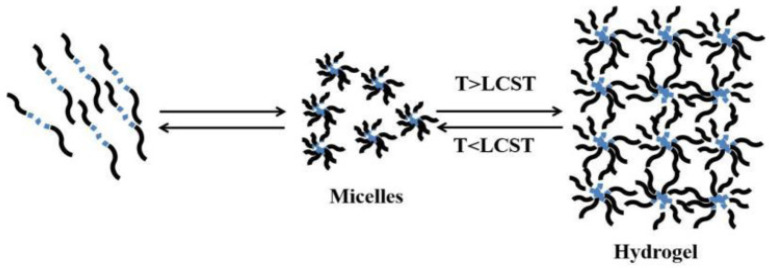
Schematic representation of the micellization and gel formation of aqueous solution. (LCST is defined as the lower critical solution temperature) [48].

Thermosensitive hydrogel is a kind of temperature-sensitive material, and the change of ambient temperature changes its physical state, resulting in the change of the sol-gel state. When stimulated by temperature, high-molecular polymers with temperature sensitivity change from a dispersed micelle state to a dense 3D network structure [48]. Phase separation takes place when the polymer solution is above or below a specific temperature, known as the critical dissolution temperature (CST). This phase separation is due to the hydrophobic effect between polymer chains, leading to polymer self-assembly and aggregation in an aqueous solution to form a hydrogel, as shown in Figure 3 [48].

**Figure 4:**
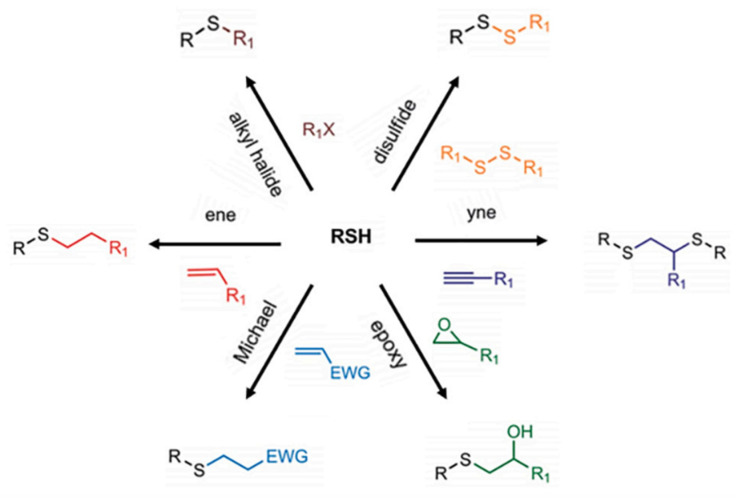
Overview of cross-linking reactions involving thiols groups [51].

Thiolated hydrogels are another type of in situ gelling hydrogels. Thiolated polymers are biocompatible synthetic polymers with free and exposed thiol groups on the surface of the polymeric backbone, covalently attached by different synthetic routes [49]. They mimic, in many ways, endogenous polymers such as proteins that also exhibit thiol substructures because of cysteine subdomains [49]. Hence, thiolated hydrogels are synthesized using thiomers such as thiolated hyaluronic acid, chitosan, cyclodextrin, poly(ethylene glycol), and dextran that are cross-linked via their thiol substructures. Additionally, thiolated polymers can form disulfide bonds, in particular, with cysteine-rich proteins such as mucins or keratins providing a firm adhesion to numerous biological surfaces [50]. These thiol groups are also beneficial in order to provide a cross-linking via disulfide bonds within their own structure forming stable 3D hydrophilic networks [50]. Furthermore, since thiol groups on the polymeric backbone of thiomers can not only react with each other but also with different other functional groups, several “click” methods such as thiol–ene/yne, Michael-type addition, and thiol–epoxy reactions, have been developed within the last decades to fabricate thiomer hydrogels. An overview about the different cross-linking reactions is provided in Figure 4 [51]. Such hydrogels are used as scaffolds for tissue engineering, regenerative medicine, diagnostics and as matrix for drug and protein delivery.

### 2.2. Advantages of Hydrogels

Hydrogels can be used as carriers for controlled drug release, providing various advantages and disadvantages in drug delivery applications. The unique physical characteristics of hydrogels have generated significant interest for their use in drug delivery applications [22]. The highly porous structure of hydrogels can be easily adjusted by manipulating the density of cross-links within the gel matrix and the hydrogels’ affinity to the surrounding aqueous environment during swelling [22]. This porous nature allows drugs to be loaded into the gel matrix, with subsequent sustained and controlled release of the drug at a rate dependent on the diffusion coefficient of small molecules or macromolecules through the gel network [22]. This allows precise dosing and prolonged therapeutic effect, which reduces the frequency of administration. Thus, the advantages of hydrogels in drug delivery are largely pharmacokinetic, especially since hydrogels can be administered directly to the site of action, such as wounds, injured tissues, or tumors, allowing for localized therapy and minimized systemic side-effects. This is achievable since they can create a depot formulation from which drugs are released gradually, sustaining a high local concentration in the adjacent tissues for an extended period, even though they are also applicable for systemic delivery [22]. Furthermore, hydrogels can protect drugs from degradation in the body, thereby improving the stability and bioavailability of the encapsulated drugs. Biodegradability and dissolution can be designed into hydrogels by allowing them to respond to specific stimuli like enzymatic, hydrolytic, or environmental (e.g., temperature, pH or electric field) triggers found in disease environments, thus enabling targeted drug delivery to the affected tissues or cells [22]. Hydrogels also possess relative deformability and can conform to the shape of the surface to which they are applied. As a result, specific hydrogels with muco- or bio-adhesive properties can offer advantages by anchoring them at the application site or by facilitating their application on non-horizontal surfaces [22]. Additionally, hydrogels generally exhibit remarkable biocompatibility and can be formulated to be non-toxic and non-immunogenic, making them suitable for use as drug-delivery systems [22]. This biocompatibility arises from the abundant water content of hydrogels and the physiochemical resemblance of hydrogels, both in composition and mechanically, to the native extracellular matrix [22].

### 2.3. Disadvantages of Hydrogels

Despite these many advantages, hydrogels also have some limitations. The low mechanical strength of many hydrogels limits their use in load-bearing applications and can lead to their premature dissolution or drifting away from the intended localized site, this fragile nature of the hydrogels also makes them difficult to handle [22]. This limitation may not be important in many conventional drug delivery applications (like subcutaneous injection); however, it is a concern revolving around the drug delivery properties of hydrogels [22]. Moreover, the quantity and homogeneity of drug loading into hydrogels could be restricted, especially when dealing with hydrophobic drugs, this could impact their utility for high-dose drug delivery [22]. The elevated water content and large pore size of most hydrogels often contributes to the relatively rapid release of drugs, over several hours to several days, which could lead to some uneven therapeutic effects [22]. Besides that, the chemical cross-linking agents used in the formulation of the hydrogel could cause toxicity. Finally, the ease of application may present challenges, although certain hydrogels are sufficiently deformable to be injected, many hydrogels are not, thus requiring surgical implantation [22]. Each of these limitations may greatly restrict the practical use of hydrogel-based drug delivery therapies within clinical settings [22].

### 2.4. Biomedical Applications of Hydrogels

**Delivery of drugs:**

Hydrogels have been extensively investigated for various drug delivery applications, including oral, transdermal, ocular, and injectable drug-delivery systems [52], as follows:

- Ocular drug delivery: The use of hydrogels as carriers in ocular drug delivery has been investigated for various conditions such as glaucoma and dry eye syndrome. These hydrogel-based systems provide a sustained release of the therapeutic agents and an improved bioavailability in the eye, thus improving patient compliance and reducing the need for frequent administrations [52]. Restasis (cyclosporine ophthalmic emulsion) is an example of a successful hydrogel-based drug-delivery system which is used for treating dry eye syndrome. Restasis is a hydrogel-based ophthalmic emulsion that delivers cyclosporine to the eye to reduce inflammation and increase tear production [53].
- Oral drug delivery: Hydrogels have been investigated as potential carriers for oral drugs especially as controlled release formulations of therapeutic agents to be used in the gastrointestinal tract [52]. They provide the encapsulated drug with protection from the harsh acidic environment of the stomach, allowing it to release its drug content in a controlled manner in the intestines, which allows for an enhanced efficacy and bioavailability [52]. An example of such a delivery system is the hydrogel-based metronidazole bio-adhesive tablet designed for oral administration. Metronidazole is an antibiotic and antiprotozoal medication, commonly used in the treatment of various bacterial and parasitic infections [54]. The tablet is designed to stick to the mucous membrane within the oral cavity (inside of the cheek or gum) [54]. The hydrogel formulation swells when it comes into contact with saliva and forms a bio adhesive layer against the mucosal surface. Metronidazole, which is embedded within the hydrogel matrix, is gradually released as the hydrogel erodes or swells [54]. As a result, this sustained release can lead to prolonged drug exposure to the mucosa, thus improving drug absorption and therapeutic effectiveness [54].
- Transdermal drug delivery: Hydrogels can offer advantages such as sustained drug release, improved patient compliance and reduced side-effects which makes them suitable to act as matrices of transdermal drug-delivery systems [52]. To ensure a consistent concentration of the drug in circulation, hydrogel based transdermal patches are designed to release drugs at a controlled rate [52]. An example of a hydrogel-based transdermal drug-delivery system is the fentanyl patch [55]. Fentanyl is a potent opioid used to manage chronic pain. The fentanyl patch is a transdermal system that consists of a hydrogel reservoir containing the drug, which adheres to the skin and releases fentanyl slowly and consistently over an extended period (typically 72 h) [55]. The hydrogel component of the patch helps maintain a constant and controlled release of the drug through the skin over time, thus providing a long-lasting pain relief [55]. Moreover, it adheres well to the skin and allows for comfort and convenience in application and wear. This method of drug delivery is useful for patients with chronic pain who require continuous pain management without frequent dosing [55]. Hence, the slow, controlled release of the medication through the hydrogel minimizes the need for repeated administration [55].
- Injectable drug delivery: The use of injectable hydrogels can allow for a localized and controlled drug delivery, especially in the treatment of diseases like cancer and diabetes. Such injectable formulations are administered using minimally invasive techniques, forming a depot at the injection site, which releases the drug in a controlled manner over an extended period [52]. An example of a hydrogel based injectable drug is Lupron Depot, which is a long-acting formulation of leuprolide acetate [56]. Leuprolide acetate is a synthetic peptide analogue of the naturally occurring gonadotropin releasing hormone (GnRH), used for the treatment of various medical conditions including prostate cancer and endometriosis [56]. Lupron Depot is formulated as a phospholipid based injectable hydrogel that releases leuprolide acetate gradually over an extended period [56]. The hydrogel system is used since it acts as a controlled release system, allowing for the sustained and controlled release of leuprolide acetate over a period of one to six months into the body, thus resulting in a persistent suppression of testosterone release and eliminates the need for frequent injections [56].

One successful hydrogel-based medication is diclofenac sodium gel. Diclofenac sodium gel is a topical medication which is commonly used to treat musculoskeletal pain and inflammation. It contains the non-steroidal anti-inflammatory drug (NSAID), diclofenac as its active ingredient. Diclofenac sodium has excellent anti-pyretic, analgesic, and anti-inflammatory effects; hence, it is used in the management of pain, osteoarthritis, rheumatoid arthritis, and ankylosing spondylitis [57]. Diclofenac sodium possesses a very short half-life (1–2 h) and is associated with a rapid and complete absorption when administered orally thus it can have undesirable side-effects, such as peptic ulcers and gastrointestinal bleeds when administered frequently [57]. As a result of the adverse drug reactions associated with oral formulations of NSAIDs, many are increasingly administered topically to reduce the risk of systemic side-effects and improve patient compliance [58]. In addition, the hydrogel-based diclofenac sodium acts as a non-invasive delivery system that is minimally disruptive to the tissues, unlike injections. In a study which tested different topical formulations of diclofenac (hydrogel 1% diclofenac sodium and two emulsion gels (1.16%/2.32% diclofenac diethylamine), it was observed that after 5 h from applying the formulations to the donor cell skin surface, the total quantity of hydrogel based diclofenac delivered through the skin was tenfold greater than that achieved with the emulsion gel 1.16% (*p* = 0.0004) and about two times the amount of the emulsion gel 2.32% (*p* = 0.022) [59]. Hence, it was concluded that the hydrogel delivery of diclofenac shows a quicker onset and higher absorption rate compared to the emulsion gel formulations which indicates that the hydrogel formulation might have a faster onset of action in the deeper tissues compared to the emulsion gel products [59]. According to the literature, diclofenac sodium topical hydrogel is formulated as follows:

- Guar gum (produces colloidal dispersions in water) and Carbopol 940 (soluble in water) were chosen as hydrophilic polymers, to help create the gel matrix and provide the desired rheological properties. While 0.1 N NaOH solution was used as a cross-linking agent [58].
- Polymeric dispersions were prepared at concentrations ranging from 0.1% to 5% separately. Then, hydrogels were fabricated by mixing different concentrations of guar gum and Carbopol 940 colloidal dispersions.
- 0.1, 0.5, 0.75, and 1% concentrations of carbopol940 colloidal dispersions were prepared using distilled water.
- Similarly, 0.1, 0.5, 0.75, and 1% concentrations of guar gum colloidal dispersions were prepared using distilled water.
- After complete dispersion, both polymer solutions were kept in the dark for 24 h for complete swelling.
- Dispersions of polymers were made using a magnetic stirrer (500 rpm).
- After dispersing Carbopol 940 in distilled water, colloidal dispersion of guar gum was added to it under magnetic stirring. The mixture was stirred until the polymer was fully hydrated and evenly distributed.
- 1% *v*/*v* isopropyl myristate and 0.0025% *w*/*v* benzalkonium chloride were added.
- Aqueous drug solution was added to the polymeric dispersion after the addition of sodium hydroxide solution, while continuously stirring to ensure uniform distribution [58].
- Finally, the remaining distilled water was added to obtain a homogenous dispersion of gel under magnetic stirring [58].

**Delivery of cells:**

The delivery of cells typically involved the injection of high-density cell suspensions into the target diseased or injured site. However, such a direct cell injection method is often associated with poor therapeutic responses due to the rapid decrease in cell viability, low or modest engraftment of transplanted cells, and limited control over cell fate due to the local environment [60]. The delivery of transplanted cells in a scaffold comprising biocompatible materials addresses these limitations by initially providing protection to the transplanted cells that can enhance survival and prolong retention at the site [61]. Hence, the use of injectable hydrogels to improve the rates of cell engraftment and survival after cell delivery could be considered. Moreover, these injectable hydrogels are considered promising soft materials that are composed of polymeric network structures and abundant water which can be injected into the body using a syringe or catheter for varying biomedical applications, such as wound healing, hemostasis, cell transplantation, and drug delivery. Injectable hydrogel-based cell delivery has many advantages:

- The ability to retain transplanted cells due to tissue adhesion.
- The ability to support cell functions as a scaffold.
- Immediate availability of isolated cells for use.
- Minimally invasive procedure involving syringe injection.

**Figure 5:**
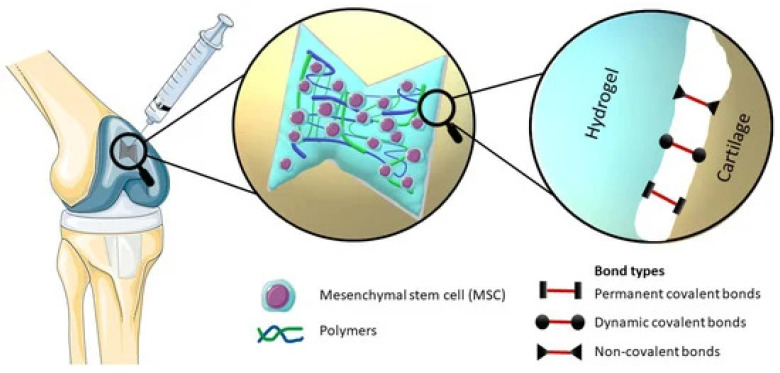
Injectable hydrogel for stem cell delivery. Illustration of an injectable hydrogel with incorporated stem cells, which effectively occupies the defect in the cartilage structure [65].

The local administration of injectable hydrogels to tissues can enhance delivery and cell retention, as well as avoid the risk of thrombosis observed with intravascular administration of cells [62]. The process of cell encapsulation using injectable hydrogels enhances cell localization to the target tissues by covering the tissue surfaces without affecting its surface geometry and anchoring them without sutures [63]. Injectable hydrogels not only deliver and retain cells as a reservoir but also provide an adhesive matrix as a scaffold; prevent anchorage-dependent cell death (anoikis); and support fundamental cell functions, such as survival, proliferation, and differentiation [64]. Moreover, injectable hydrogels do not require invasive surgical procedures, thereby reducing patient burden. Examples of hydrogel systems designed for delivering cells in a range of regenerative medicine applications are listed in Table 3 and illustrated in Figure 5.

**Table 3:** Cell delivery applications in natural and synthetic hydrogels.

	Hydrogel Material(s)	Cells Delivered	Delivery Strategy	Tissue Application	Reference
Encapsulated hydrogels	Alginate	Pancreatic islets	Laparoscopic implant of microcapsules	Diabetes	[66]
	PEG and alginate	Ovarian follicles	Encapsulated scaffold	Ovarian function	[67]
Tissue-integrating microporous hydrogels	Collagen	Autogenous chondrocytes	Porous scaffold matrix	Cartilage repair	[68]
	Gelatin	Adipose-derived stromal cells	Microporous microribbon hydrogel injected into cranial defect	Bone regeneration	[69]
Biodegradable hydrogels	Hyaluronic acid	Neural progenitor cells	Injection into stroke cavity	Neural regeneration from stroke	[70]
	Fibrin	Human embryonic stem cells	Epicardial delivery of encapsulating gel	Cardiac regeneration	[71]

**3D Cell Cultures**

Three-dimensional cell cultures provide a useful platform for the cell to grow in vitro in all directions. It can be achieved by culturing the cells on a 3D scaffold, where these in vivo cells get embedded into the extracellular matrix (ECM) to form a 3D structure. Hydrogels possessing a 3D structure and a hydrophilic polymer network are capable of absorbing water, in addition to biological fluids. Thus, they can construct a soft and wet 3D structure that resembles the ECM, making it available to encapsulate cells.

As hydrogels can comprise natural, synthetic, and semisynthetic polymers, they can provide distinct biochemical, physical, and mechanical properties for the 3D cell culture. Natural hydrogels can support cell activity for 3D cell cultures, since they have good biocompatibility, endogenous factors, and the similar viscoelasticity and fibrils to the ECM. Table 4 describes the recent application of these hydrogels in 3D cell culture.

Gene Delivery through Hydrogels

Hydrogel-based nonviral gene delivery is an effective approach in regenerative medicine, particularly for treating musculoskeletal, cardiovascular, and neural tissue disorders, as well as in wound healing [84]. Due to their capability to mimic the properties of the extracellular matrix, hydrogels can improve the survival, differentiation, and integration of the host cells. Moreover, these systems allow for a minimally invasive administration that enables the precise and controlled delivery of specific gene sequences to target tissue cells. By either overexpressing or silencing the original gene, these hydrogels can enhance natural repair mechanisms, leading to the desired therapeutic outcomes [84].

In an effort to control the release kinetics and preserve the activity of therapeutic biomolecules, hydrogels have been widely investigated as gene delivery systems. Various hydrogel systems based on natural polymers such as alginate, cellulose, chitosan, collagen, dextran, fibrin, pullulan, gelatin, hyaluronic acid, or synthetic ones as polyethylene glycol, poly(N-isopropylacrylamide), polyurethane, or poly(organophosphazene) have been studied as delivery systems of therapeutic nucleic acid (NA) molecules in various tissue engineering approaches, as shown in Table 5.

### 2.5. Hydrogels for Antimicrobial Use

Microbial infections are serious health problems, especially those related to impaired wound healing and biomedical implant failure, which is why different strategies were developed to create materials with antimicrobial activity that would prevent or treat infections occurring at wound, implant, and device insertion sites [91]. Due to the inherent biocompatibility of many hydrogels, they have the capability to serve as a convenient foundation for creating selectively active antimicrobial materials [91]. Antimicrobial hydrogels can be obtained by the encapsulation or covalent immobilization of known antimicrobial agents [91]. Some of the recently developed antimicrobial hydrogels are discussed as follows.

A recent study has focused on the development of a flexible topical hydrogel patch loaded with neomycin to enhance wound healing [92]. The hydrogel based transdermal patch containing neomycin was created using sodium alginate (SA) and hydroxyethyl cellulose (HEC) as polymers for the gel matrix, and it was formulated using the free radical polymerization method which occurred in an aqueous environment, by using acrylic acid (AA) and N,N’-methylenebisacrylamide (MBA) [92]. The study analyzed the hydrogel’s properties, including pH sensitivity, sol–gel behavior, and swelling patterns [92]. Scanning Electron Microscopy (SEM) assay displayed the porous structure of the hydrogel’s polymer matrix, leading to an increased capacity to absorb fluids, while Fourier-transform infrared spectroscopy showed the formation of a new polymeric network [92]. Thermal analysis indicated enhanced stability in the fabricated patches [92]. Additionally, experiments conducted on excised rabbit’s skin confirmed the retention of the drug within the skin layers [92]. As a result, the neomycin hydrogel patch effectively facilitated the healing of wounds inflicted on the rabbit skin, and the formulation did not induce any irritation on the intact skin [92]. Hence, the neomycin hydrogel patches present a promising option for controlled delivery, aiding efficient wound healing [92].

An article released by Zakaria et al. focused on evaluating the antibacterial activity and in vivo performance of newly developed hydrogels containing cefotaxime, to treat bacterial skin and soft tissue infections more efficiently [93]. The topical hydrogels were formulated using natural and synthetic polymers, namely, Carbopol 934 (C934), Hydroxypropyl Methylcellulose 4000 (HPMC 4000), Carboxymethylcellulose Sodium (Na CMC), Pectin (PEC), Xanthan Gum (XG), or Guar Gum (GG) [93]. Different concentrations of various polymers (gelling agents) were postulated to prepare 13 different formulas. These drug-loaded hydrogels were formulated by dispersing the gelling agents slowly in an aqueous-based solution containing Cefotaxime (1% *w*/*w*), PG (5% *w*/*w*), and sodium benzoate (0.25% *w*/*w*), with the help of an overhead mechanical stirrer at a moderate speed. In vitro and in vivo antibacterial activities of Cefotaxime were studied against wound pathogens such as, S. aureus, E. coli, and P. aeruginosa, using either pure drug or Fucidin cream as control [93]. Among all gel formulations, Cefotaxime gel (F13) prepared from C934 (2%) gel reservoir proved to be the formula of choice, since it provides better spreadability compared to F1 (XG) or F11 (HPMC) [93]. Furthermore, the release of the drug from hydrogel F13 was slower and sustained for 8 h. In addition, F13 showed the largest inhibition zone and highest antibacterial activity among other formulations [93]. Additionally, against standard P. aeruginosa there was a decrease in the number of survivors of more than 6-log reduction compared to the control by the application of F1 and F13. Histological analysis demonstrated that after a single treatment with F13 gel formulation, a noticeable reduction in microbial bioburden occurred in case of both Gram-positive and Gram-negative bacterial isolates [93].

### 2.6. Summary of Hydrogels Finding

- Hydrogels are composed of cross-linked hydrophilic polymeric networks that are capable of absorbing and retaining significant amounts of water or biological fluids.
- Several strategies and procedures are used to formulate hydrogels, depending on the specific requirements of the intended pharmaceutical application.
- Hydrogel formulation process involves selecting a suitable polymer, cross-linking agents, and additives, as well as determining the appropriate processing conditions.
- The use of hydrogels in drug delivery has several advantages, including controlled drug release, localized therapy, protection of drugs from degradation, conformability, and biocompatibility.
- Hydrogels have several limitations, including low mechanical strength, limited drug loading, rapid drug release, toxicity of cross-linking agents and application challenges.
- Each of these limitations could significantly limit the application of hydrogel-based medication-delivery treatments in the clinical contexts.
- Hydrogels have various applications in the drug delivery field, including oral, transdermal, ocular, and injectable drug-delivery systems.
- One example of a successful hydrogel-based medication is diclofenac sodium gel. This topical medication is widely used to alleviate musculoskeletal pain and inflammation.
- Due to the inherent biocompatibility of many hydrogels, they serve as a convenient foundation for creating selectively active antimicrobial materials.
- Antimicrobial hydrogels can be obtained by the encapsulation or covalent immobilization of known antimicrobial agents.

## 3. Aerogels

### 3.1. General Overview

Having been introduced in 1931, aerogels were first described by S. S. Kistler as having “new physical properties” that are of “unusual interest” [94]. His first preparations of synthetic silica aerogels resulted in opalescent, low-density gels with glassy fractures. He additionally reported their unique ability to emit a metallic ring when dropped in small pieces [94]. From the first recounted experimental procedure by Kistler until today, not much has changed in the basic approach to how aerogels are formulated, besides the sol–gel and the drying techniques, which are much simpler now [95]. Recently, more research is being conducted on the use of these gels in drug delivery, with numerous papers having outlined the various methodologies in which drugs could be loaded and released from aerogels, as well as some in vitro trial results from their advances. It is relevant to mention that various aspects must be controlled during formulation to create the optimum tailor-made gel vehicle for the delivery of a designated drug. If aerogel nanoparticles are used, then poor pharmacokinetics, lack of selectivity, unfavorable biodistribution, and other challenges exhibited with free drug delivery could be overcome [96]. Factors such as the materials used for the aerogel network, the pore sizes, and the solvent used during the sol–gel step can all affect the final product differently. This section hopes to address some common methods for the formulation of aerogels with emphasis on their relevance in drug loading and delivery, while also noting any recent advancements in relation to their use in pharmaceutical sciences.

### 3.2. Aerogel Formulation

As mentioned previously, the production of aerogel typically requires three major steps which are sol–gel formation, aging, and finally drying [14]. The process typically begins by converting the gel precursors into hydrogels or alcogels using chemical cross-linkers, or alternatively by physically altering their surrounding temperature or pH [95]. To complete these reactions, the gels are then placed in a combined solution of water and alcohol (an aging solution) [96]. The formed gels are then washed or subjected to solvent exchange, where the solvent used in the formation of the network during the sol–gel stage is switched out to another that can be easily evacuated from the pores. After that, the formed gels go through the drying stage, where they are subjected to an appropriate drying technique in order to void the pores of any solvent inside them, while maintaining the outer network structure [95].

The starting mixture used to develop these gels can be formed into different shapes and sizes, allowing for aerogels to be formulated into beads, spheres, powders, sheets, coatings, and other necessary forms that are required to meet the conditions of their application [14]. Typically, in order to be utilized as drug carriers, aerogels are preferred to be spherical (microspheres). This is attributed to the idea that a rounder shape would allow them to have an increase in flowability, better reproducibility, simplicity in handling, an increase in surface area, and, finally, a lack of sharp edges, thus lowering their chance of provoking an inflammatory response in the body [14,95]. Smirnova et al. described that the use of microparticulate aerogels as drug carriers offers a great surface area which would help in achieving quicker drug loading, release, and absorption rates in the body [97].

#### 3.2.1. Sol–Gel

Also known as wet chemistry, sol–gel is a technique entailing the formation of a three-dimensional lattice structure through the processes of solution and gelation [98,99]. The first step involves the formation of a sol (a colloidal suspension), which is a type of solution where colloidal nanoparticle solutes undergo hydrolysis and condensation in order to be suspended in a solvent. Following that is gelation, where the nanoparticles in the sol are subjected to cross-linking, which allows for them to approach a point of gelation and to form the three-dimensional network. To form cross-links during gelation, two methods can be utilized, which are chemical cross-linking and physical cross-linking [16,100]. The chemical cross-linking process involves the formation of strong covalent bonds through the use of chemical agents, such as N,N-methylenebisacrylamide (MBA), or by altering the surrounding pH or temperature, allowing the particles in the sol to agglomerate into their lattice form. Physical cross-linking, on the other hand, is achieved by chain entanglement, by electrostatic interactions, or through the formation of weak physical bonds, such as hydrogen bonds and van der Waal forces [100].

The time required for the gelation process may vary based on the concentration of the material used to form the network, the type of solvent, the type of precursors, and the pH and temperature under which the gelation is happening [14,100]. Zhongming Liu et.al reported that the rate of gelation obtained by chemical cross-linking is more rapid resulting in more stable gels in comparison to the gels formed by physical cross-linking [100]. Having said that, since the chemically formed gels are stronger, they often exhibit irreversible bonding at body temperatures, which may hinder their degradation [16]. Nonetheless, even if different precursors or procedures are used, all formed aerogels must undergo the sol–gel process [95].

Following the sol–gel process, a wet gel (hydrogel/alcogel) is formed. However, if hydrogels are formed and subjected to drying by evaporation, the liquid in the pores would be ejected resulting in a xerogel with a smaller and possibly collapsed structure [98]. In order to combat this shrinkage and prevent the gel from cracking, some steps such as aging; solvent exchange; and various drying methods, such as supercritical drying, are implemented to keep the original structure of the gels.

In the case of microspherical aerogels, it is relevant to note that, during their wet chemical synthesis process, the dispersed aqueous sol is stirred vigorously into the continuous oil phase, forming an emulsion and allowing for gelation of the sol to occur [14]. Here, the size of the microspheres is affected by the ratio of the dispersed phase to the continuous phase, as well as by the use of surfactants, the concentration of precursors, and, finally, the agitation experienced by the microspheres [14]. Larger spheres (beads) can also be achieved through having the precursors dropped into the cross-linking solution using a syringe in order to achieve gelation. In this case, the size of the spheres is dependent on the size of the syringe nozzle. Overall, the formed gels are then filtered through centrifugation or simple filtration and are then washed and dried appropriately [14].

#### 3.2.2. Aging

After the wet gel formation, some remaining unreacted precursors from the sol–gel step are still in the pores. For that reason, aging, which is another essential step in aerogel manufacturing, is utilized in aiding the remaining precursors in undergoing hydrolysis and condensation reactions [96]. Additionally, aging plays a role in enhancing the mechanical strength of the gels to result in structures that are compact and that can withstand damage or shrinkage [95,96]. The process involves placing the wet gels in an aging solution, which can be one of water and alcohol [96] or it can be the initial sol from the previous step; nonetheless, the aging process can take from a few hours up to a few days [95,101]. One significant dissolution–precipitation phenomenon that occurs during aging is known as “Ostwald ripening.” In this process, smaller aggregates dissolute into the solution and precipitate on larger aggregates allowing them to grow in size [102,103]. Maleki et al. described the process as the coarsening of the gel network through the movement of particles from areas that are thermodynamically unfavored onto ones which are more thermodynamically stable. This movement will result in the pores of the gel expanding, leading to a diminished surface area [95]. Nonetheless, the thickening will result in the network being stronger, as it will now shrink less when subjected to the capillary forces of drying [95,103]. Iswar et al. mentioned a number of other ways in which the network could be further strengthened during aging, such as an increase in temperature and a prolonged reaction time. A greater temperature would allow for aging to occur at improved kinetics, while an increase in the time required for aging would allow for more hydrolysis and condensation reactions to take place [103].

#### 3.2.3. Solvent Exchange

As mentioned previously, the water in the pores of the formed hydrogels creates a significant complication when attempting to form aerogels. Regardless of whether supercritical drying were to be used alone without the utilization of solvent exchange, due to the low affinity of water to the supercritical carbon dioxide (scCO_2_), even if it is found in small amounts, drastic alterations can occur in the network pores [16]. For instance, any water left in the gel pores before supercritical drying will remain there after the drying process resulting in a dense and opaque gel [101,104]. For that reason, the main aim of performing solvent exchange is to convert any formed hydrogels into alcogels by placing them into a solvent of alcohol so it could replace the water in the pores and remove any present impurities [16,96]. After sol–gel and aging, the gels are placed in their new solvent directly or gradually in a sequential process where the water-to-new-solvent concentration is increased each time [16]. García-González et al. mentioned that, typically, acetone and alcohol, which are highly soluble in CO_2_, are used to perform the solvent-exchange process. They also highlighted some important factors which should be considered when choosing the solvent, which are that (a) the new solvent should be soluble in the original solvent used to make the gels (water), (b) it should be approved in pharmaceutical manufacturing, and (c) the new chosen solvent should not be able to dissolve the network structure of the gel [16].

Mehling et al. reported that, during their research on polysaccharide aerogels, shrinkage was observed during the solvent-exchange stage, especially for alginate hydrogels. In their experiment a gradual replacement of the original solvent (water) was carried out, and the shrinkage of the alginate gels was then attributed to the idea that, as the ethanol concentration was increased, the surface tension and the capillary forces both decreased resulting in volume loss [105]. Other papers reported similar results when subjecting biopolymer gels to solvent exchange [106,107,108]. One way in which shrinkage can be reduced, as previously observed by Cardea et al. in a water-to-acetone exchange experiment, is through the use of lower temperatures as a result of delayed kinetics or substitution in gels at stable circumstances [109].

#### 3.2.4. Drying

The final step in the formulation of aerogels is one which tackles a huge challenge in their production. In order to form an aerogel and for it to be different from the other available gels in the market, the pores need to be voided of the formulating solvents, without resulting in the collapse of the nanoporous network.

##### Freeze Drying

García-González et al. discussed how the use of drying techniques such as freeze drying could result in the formation of a cryogel with unavoidable damages and cracks [16]. Since freeze drying involves rapid cooling of the solvent in the pores pursued by sublimation, liquid–solid forces and liquid–vapor tensions will allow for the pores to shrink and for solid–solid interactions to form [12]. Some disadvantages associated with the use of freeze drying is the need to stabilize the network further through increasing the aging duration, or that the presenting solvent in the pores may crystallize and cause damage to the network [110,111]. Additionally, if hydrogels were subjected to freeze drying, the water in their pores would expand during the process affecting the gel network [12]. Nonetheless, these disadvantages may be overcome with the use of cryoprotectants, such as trehalose with polyethylene glycol (PEG), or by using cosolvents, such as tert-butyl alcohol, to minimize the surface tension [12].

##### Supercritical Drying

Supercritical drying on the other hand is another method which provides a solution to some of the problems experienced with the previously mentioned drying techniques. This method requires the use of critical conditions as a way to eliminate the formation of a liquid–vapor interface, preventing the creation of a meniscus, and therefore minimizing the effects of capillary tension on the shrinkage of the network [101]. As mentioned before, this is preferably performed on an alcogel rather than a hydrogel to avoid any water from remaining in the gel after drying. One other importance to void the gels of water was highlighted by Kayser et al. in their study on chitosan gels; they stated that if any water remains in the pores, carbonic acid will result from the interaction of water and critical carbon dioxide, leading to the dissolution of the gels [112].

Typically, there are two ways in which supercritical drying can be carried out: dynamic supercritical drying and static supercritical drying [16]. The main difference is that the dynamic process involves loading the wet gels into an autoclave or an extractor with a continuous flow of liquid scCO_2_, as the pressure then accumulates, turning it into gaseous CO_2_ that is enriched with the alcoholic solvent from the gels, and ultimately depressurization is employed to form the aerogel. The static process on the other hand requires the drying of the gels with scCO_2_ but in batches of 6–12 h. After that another flow of liquid scCO_2_ is used to replace the alcohol infused CO_2_ gas in the extractor with a newer one, this then can be repeated for as many cycles as necessary ending it with the release of pressure to the atmospheric one and the decrease in temperature to that of room temperature to obtain the final aerogels [16].

Supercritical drying can also be achieved by exposing the solvent in the gel to conditions beyond its critical point; however, this is not the best option for solvents which are easily flammable, such as ethanol or acetone [16]. It is relevant to note that the pore structure, surface area, and density of the final aerogel produced by supercritical drying may be impacted by factors such as temperature, pressure, drying time, and depressurization rate [101]. Organic gels such as those of polysaccharide origin would risk degradation at the high temperatures needed to surpass the critical point [16]. For instance, polysaccharide aerogels require the use of low temperatures of 310–330 K when processing with scCO_2_ to prevent any conformational changes or interactions within the molecular chains [113].

A positive of working with this drying technology is that it allows for the use of material which would otherwise not be used in hydrogels such as poorly water-soluble substances, as well as the ability to integrate an array of solvents in the production [12]. Nonetheless, the requirement for using high pressure in this process is the use of sturdy equipment, which can often be costly. Over the years, industrial utilization of scCO_2_ has aided in a considerable cost reduction [17]. Nevertheless, this technique proves to be the most effective in maintaining the aerogel structure after drying.

In a noticeable study conducted by López-Iglesias et al., chitosan aerogel beads that are loaded with vancomycin were produced by the typical sol–gel, aging, and solvent-exchange steps followed by drying using supercritical techniques. The final aerogels, which were a result of supercritical drying, exhibited less shrinkage in comparison to those freeze-dried or placed in an oven. Furthermore, the supercritically dried gels had a large surface area and a porosity of around 96.8%, which the authors highlighted was better than the results exhibited by the evaporated and frozen gels, as they were not able to preserve the nanoporous network in their gel. Overall, the scCO_2_ gels were able to absorb large amounts of fluid that could maintain moisture at a wound site, as vancomycin rapidly exerted its therapeutic local antibiotic effect to prevent infections, making them a promising vehicle for future formulations in wound healing [114].

##### Ambient Pressure Drying (APD)

Due to the exorbitant cost and risk associated with scaling, and the equipment needed in supercritical drying, one other affordable drying technique considered is ambient pressure drying (APD) [111,115,116]. This technique demands the use of modifications, namely silation of the gel surface to preserve its structure [111]. The wet gels are then dried by subjecting the solvent in the pores to evaporation at room temperature or any other temperature up to 200 °C, under ambient pressures [117]. Without applying surface modifications, if the formed hydrogels are left to air dry, their structure would collapse forming shrunken xerogels, rendering this process counterproductive especially in hydrophilic gels [111,116,118]. The wet gels are then dried by subjecting the solvent in the pores to evaporation at room temperature or any other temperature up to 200 °C, under ambient pressures [117]. Without applying surface modifications, if the formed hydrogels are left to air-dry, their structure collapses, forming shrunken xerogels, rendering this process counterproductive, especially in hydrophilic gels [116,118]. This is mainly due to the idea that, as water evaporates from the pores, the liquid–vapor interface will result in the formation of a surface tension meniscus between the liquid and the solid walls of the pores; this, in turn, results in a capillary tension gradient, creating a pressure which results in a contractive pull as the water evaporates, eventually leading to the collapse of said pores [98,101]. It is worth noting that the size of the gel pores affects the rate of evaporation since larger pores are seen to empty quicker compared to smaller pores due to the presence of high vapor pressure, as all the menisci have the same curvature at the same pressure [115,119]. Nonetheless, in some circumstances, smaller pores prove to be easier to dry than larger ones under the theory of cavitation [116].

In 1988, Scherer outlined the gel-drying phenomenon in an intriguing way [119]. Since gels are made of a continuous solid lattice surrounded by a continuous liquid phase, the evaporation of that liquid phase will allow whatever liquid remaining in the pores to be stretched over the exposed solid network, due to the energy of the solid–vapor interface being stronger than that of the solid–liquid interface. This stretching will then result in the formation of “tensile stress” in the liquid phase, which then imposes “compressive stress” on the solid phase. Due to that, the compliant gel network will experience shrinkage at the same rate as the evaporation occurring simultaneously, resulting in the collapse of that network under the liquid surface as the liquid–vapor meniscus remains at the gel’s exterior surface [119]. Scherer then mentioned that, as evaporation continues, concurrent aging and further shrinking of the gel cause the solid network particles to become more tightly packed, resulting in a stiffer gel. Shrinking and stiffening of the gel then proceeds, increasing the pressure at the surface where the meniscus is until it rises to its maximum curvature, putting capillary pressure on the network and halting the shrinkage process at the “leatherhard point” or critical point, which, if abrupt, can result in a stress increase that would crack the gel [110,119]. Ultimately, the meniscus starts receding into the solid network, and the surface temperature rises to ambient.

In order to make use of the more affordable ambient drying method without subjecting the aerogel structure to deformation or fractures, the capillary stress would need to be minimized, and the structure would have to be hydrophobized. Much research has gone into developing non-silica aerogels out of ambient pressure drying, yet it seems that, in most cases, silica aerogels produced from ambient drying are the closest in properties to those supercritically dried [120]. A few of the methods recommended alongside the gel synthesis to maintain the aerogel structure include strengthening the network, performing surface treatment, or applying both for a “spring-back” phenomenon after drying [117]. To achieve said methods, two routes can be followed: either the alkoxysilane route or the waterglass route. Silylation can then be carried out to modify the inner surface of the gels and reduce the contact angle between the pore walls and their contained liquid [111].

Deshpande et al. developed a patented surface modification process addressing the bonds that form on the gel surface as a result of condensation reactions of the surface groups with water. These bonds prevent gel expansion after drying, and they maintain the collapsed structure of the wet gels. In this invention, surface modification compounds bind to the surface of the surface groups forming unreactive MR*_x_* groups. These groups then increase the contact angle of the meniscus in the pores and prevent condensation from occurring [121].

Rao et al. synthesized hydrophobic trimethylethoxysilane/tetraethoxysilane (TMES/TEOS)-based silica aerogels and discussed the correlation between TMES/TEOS and network strengthening. They described the TMES/TEOS molar ratio as (A) and explained that, as it increases, the volume of shrinkage of the gel decreases and then increases. This was further clarified by the loss of H from the -OH by the TMES group, minimizing shrinkage and allowing the cross-linkage in the network to remain strong. After a certain point, a high A value will cause steric crowding resulting in shrinkage. One important thing to note is that, as A increases, the silica aerogel becomes more hydrophobic, showing an increase in its contact angle [122].

Hwang et al. formed silica aerogels by preparing wet gels from a waterglass solution (sodium silicate), followed by solvent exchange and surface modification using a solution of isopropyl alcohol, TMCS (trimethylchlorosilane), and n-Hexane. The gels were then left in this solution for one day under 60 °C and then dried at room temperature for 3 days to control evaporation in an n-Hexane atmosphere, where they exhibited a 50% shrinkage. Finally, the gels were heated at 50 °C for 1 h and at 230 °C for another hour in air, where their volume was restored up to 95% of the original volume. This decrease in and restoration of volume is what is often described as a “spring-back” effect [123].

### 3.3. Drug Loading in Aerogels

**Figure 6:**
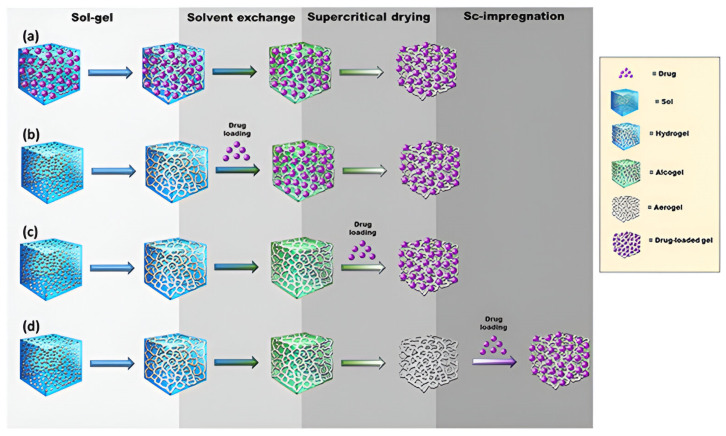
Aerogel drug-loading techniques: (**a**) drug loaded into the precursor used during the sol–gel phase, (**b**) drug loaded into the gel during solvent exchange, (**c**) drug loaded during the supercritical drying phase, and (**d**) drug loaded after the gel is formed [12].

Several strategies are utilized to load drugs into aerogels based on their solubility and stability (Figure 6):

(a) The first method is one used for drugs which have a limited solubility in both the organic solvent from the solvent exchange step and the scCO_2_ from the drying stage, as well as those which are stable in normal gelling conditions. The loading of these drugs consists of adding them to the precursor solution, where they are employed to prevent the drug from being extracted prematurely [12]. One disadvantage associated with this method is that the drugs may react with the precursor or other reagents used during the gel formation [98]. Things like temperature and the pH of the sol need to be considered during this stage [14]. A reason why this method is chosen, however, is due to its simplicity and flexibility with different compounds [14].
(b) Drugs which are soluble in organic solvents but not scCO_2_ can be incorporated into the gel after it is formed. Here, the gel is soaked in the drug containing loading solution and then subjected to drying, where the solvent is evaporated, leaving the drug to precipitate in the gel pores. One drawback to this method is that the loading process can be slow and incomplete, as it takes time for the drug to diffuse into the inner matrix of the gel. This leaves the outer layer of the gel more concentrated than that of the internal network. Nonetheless, this method allows for drug crystallization to occur [12].
(c) The third method is for drugs which are soluble in scCO_2_ and in common solvents which can be introduced into the gel during the supercritical drying stage [12]. This helps to reduce the number of steps during processing, as well as bypass the need to use elevated temperatures or organic solvents to load the drugs [14]. A few other advantages associated with this method include enhanced drug solubility and diffusion into the aerogel matrix, as well as the ability to preserve its structure. This method can additionally help evade the need for purification, and when it comes to spherical aerogels, it allows for a more homogenous drug distribution [14].
(d) The last technique involves forming the aerogel first and then loading the drug onto it through scCO_2_ impregnation, which can be performed as a one-pot process. This method is also known as adsorption precipitation, and it helps prevent the partial dissolution or deswelling experienced by the aerogels when immersed in a solvent [12]. Additionally, besides the scCO_2_ being non-toxic, it can evaporate rapidly out of the gel, eliminating the need for any additional solvent removal which may result in the network pores collapsing [12,14].

### 3.4. Drug Release from Aerogels

Besides knowing how to load drugs into aerogels, its relevant to understand how these drugs get released from the gel matrix. The two mechanisms governing this process are drug dissolution and drug transport from the matrix to the dissolution media [12]. These mechanisms can additionally be affected by physiochemical properties, such as (a) the hydration of the drug and the matrix, (b) the interactions between the drug molecules and the network, and (c) the mass drug transport occurring after hydration [12].

During drug delivery, the hydration of the aerogel and the dissolution of the loaded drug typically start on the surface of the network and then migrate through the pores toward the center [12]. Here, the hydrophilicity of both the network and the drug plays a role in the rate at which the drugs dissolute out of the gel. Rapid hydration and dissolution occur when both the network and the drug are hydrophilic. The rate of this process is only affected by the mass transport of the drug from the outer matrix toward the dissolution solvent. Alternatively, having a hydrophobic active ingredient loaded into the aerogels results in a slower drug dissolution out of the matrix [12].

When the gel network is highly hydrophilic, the rapid hydration of the network backbone results in the development of a thick hydration later which forces the loaded drug to be displaced, accelerating the dissolution rate [12]. This is often observed in aerogels made of polysaccharide and silica. In hydrophilic networks which do not swell, mass transport remains unopposed due to their highly open porous structure. On the other hand, hydrophobic networks, such as those made of biopolymers and hydrophobized silica, can result in delayed drug dissolution due to the considerable time hydration requires [12].

Inorganic oxide, as well as cross-linked (bio)polymer matrices, can erode rapidly and spontaneously in water to highly porous microparticles [12]. This results in a rapid release of the drug from the gel. As previously, if the matrix being eroded is hydrophobic, then the rate of drug dissolution is hindered. When carbohydrate and protein gels achieve complete hydration, swelling and rearrangement occur, forming a hydrogel-like network. This can result in the drug being trapped within the matrix, delaying drug diffusion out of the pores. Moreover, the speed at which they swell could impact the rate at which the active ingredient dissolves. Thus, a slower hydrogel formation gives the drugs a chance to diffuse out of the gel before their entrapment within the collapsed matrix [12].

Another factor that can also play a role in drug dissolution is the chemical properties of the medium which the drug is released into. Additionally, combining both organic and inorganic properties of these aerogels can help in designing the desired properties for aerogels as drug carriers [12]. Factors such as surface area are important in controlling dissolution rate and drug absorption rate in the body. Esquivel-Castro et al. (2019) reported that drug delivery over long periods is possible in aerogels, as they possess a large surface area, which reduces their drug dissolution rate [98]. Furthermore, placing hydrophilic gels in an aqueous solution allows the hydrophobic drugs loaded in them to experience rapid dissolution. This is due to the generation of surface tension inside the pores, eventually leading to the collapse of the gel network. The opposite is true for the hydrophobic gels [98]. Overall, there has to be a high affinity between the aerogel matrix and the drug in order to increase drug loading, dissolution rate, and bioavailability [124]. García-González et al. (2021) discuss drug release in more detail [12].

### 3.5. Advantages of Aerogels

The use of aerogels in drug delivery allows for several benefits. First, the ability to develop various dosage forms, such as solid, liquid, and gaseous, enables the pharmacokinetic properties of known APIs to be enhanced [124]. Second, the high porosity of these gels promotes their ability to absorb liquids; this, in turn, allows the gels to be excellent in terms of wound healing, as they can absorb exudates to preserve moisture levels at the site of injury while also maintaining drug release [125]. The low density which these aerogels possess has allowed them to be an interest in pulmonary drug administration [126,127,128]. Additionally, it has been reported in the literature that drug-loaded aerogel microparticles can show drug dissolution that is two-to-three times quicker than that of regular solid particles, thus allowing for a more rapid absorption in the bloodstream [129]. Moreover, the recent development and ease of drug loading into aerogels allow for simpler and improved production under good manufacturing practices [12]. Though not used therapeutically, aerogels made of polyimide have been reported to be relatively strong, as they have the capability to support the weight of a car [130].

### 3.6. Disadvantages of Aerogels

With all of these advantages, aerogels still have some limitations. One reported problem is that, when administered to the body, the broad surface area of the media which the gels will encounter may result in quick and uncontrolled drug release. Nonetheless, this can be controlled by regulating the degree of cross-linking and coating of the matrix [12]. For aerogels made of silica, brittleness and sensitivity to low pressures is a reported issue [131]. Cellulose aerogels have also been documented for fragility as their internal structure can be damaged through adsorption when used for oil/water spills [132]. Moisture sensitivity has also played a factor in aerogel limitation. Moreover, the shrinkage of these gels at high temperatures has been documented. Further limitations are discussed in the proceeding sections.

### 3.7. Application of Aerogels

Over the past several years, aerogels have been involved in various fields. Gels made of silica have been the most utilized due to their properties. In aeronautics, The National Aeronautics and Space Administration (NASA) employed silica-based aerogels in the Mars rover because of their insulative properties [133]. NASA has also turned to aerogels in order to maintain the cryogenic temperature of rocket fuel [134], as well as to capture high-velocity particles during missions [135].

In an environmental aspect, the use of cellulose aerogels to control oil spills in water is also documented. This is because of their biodegradability, high porosity, and large surface area [132,135,136]. Carbon aerogels have also been used for such applications where they showed recyclability after 10 cycles of absorption by maintaining up to 85% of their absorption capabilities [137]. On an ecological scale, heavy metals that get emitted regularly from industrial facilities into sewage water and eventually reach aquatic environments require filtration [138,139]. Here, porous and hydrophobic carbon aerogels have been utilized to separate these contaminants [138,139]. In the management of liquid nuclear waste, silver-functionalized silica aerogels could be utilized in order to capture radioiodine [140].

One other common application for aerogels is as catalysts. Current practices utilize platinum-based electrocatalysts in fuel cell oxygen reduction reactions (ORRs); however, their cost and stability create a limit to their applicability [141]. Research concerning ORR has been examining the use of loaded graphene aerogels as improved catalysts [142,143]. Reduced graphene oxide-based aerogels have also attracted attention as photocatalysts due to their surface area, porosity, light weight, and surface wettability [144]. Polysaccharide aerogel catalysts, on the other hand, are also of interest due to their ability to overcome the hindered diffusion created by the limited surface area present in the normally used xerogel and lyophilized solid polysaccharide catalysts [145].

**Figure 7:**
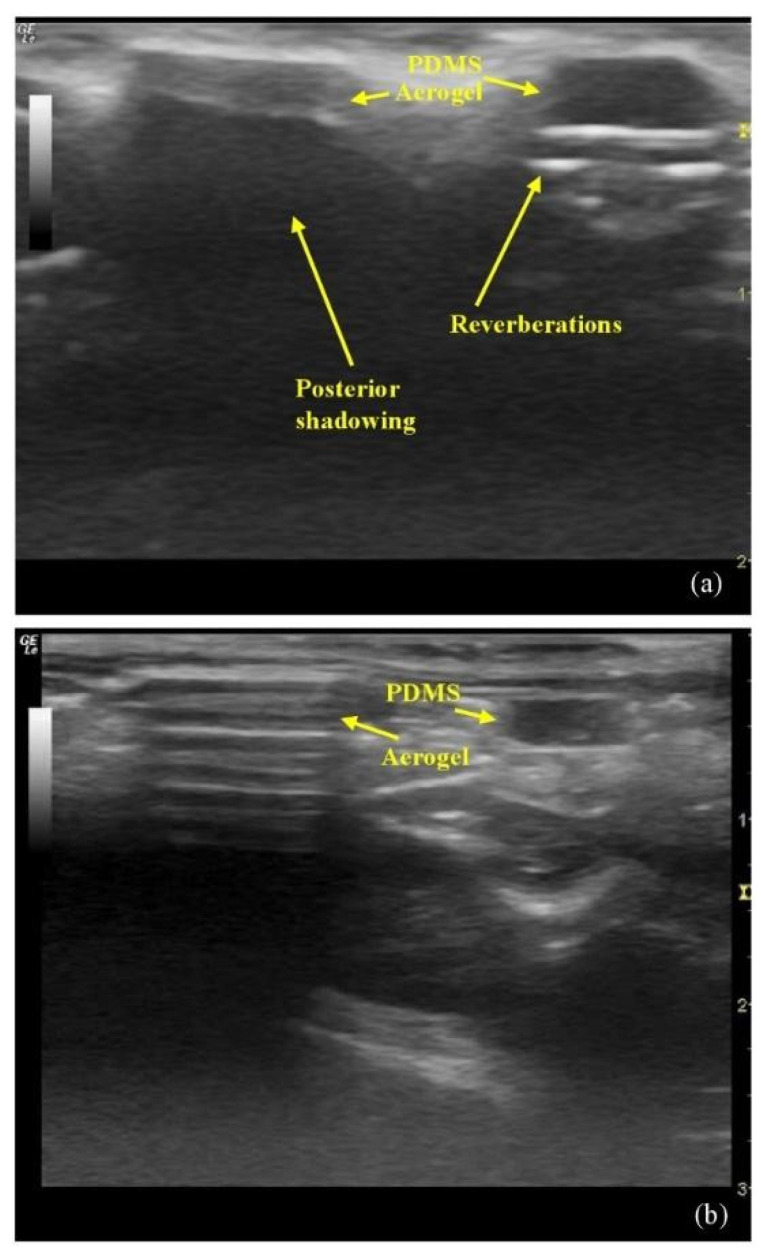
Ultrasonic images showing aerogel and polydimethylsiloxane (PDMS) (**a**) subcutaneous and (**b**) submuscular implants in a Sprague-Dawley rat [147].

In biomedicine, aerogels have gained an increased fascination due to their exceptional properties. Plenty of research has documented the biocompatibility of aerogels using in vitro [146] and in vivo studies [147]. In vitro studies using polyurea-cross-linked silica aerogel that were treated with a growth-promoting coating have demonstrated the ability to allow dorsal root ganglia neurons to grow and differentiate [146]. In vivo ultrasound response studies utilizing the same cross-linked gel implants were conducted in rats and human cadavers (Figure 7) [147]. They documented that the implants had maintained their geometrical identity, as well as their possibility to not interfere with the imaging of skeletal structures. Eventually it was concluded that aerogel based materials have a strong potential in biomedical applications [147].

**Figure 8:**
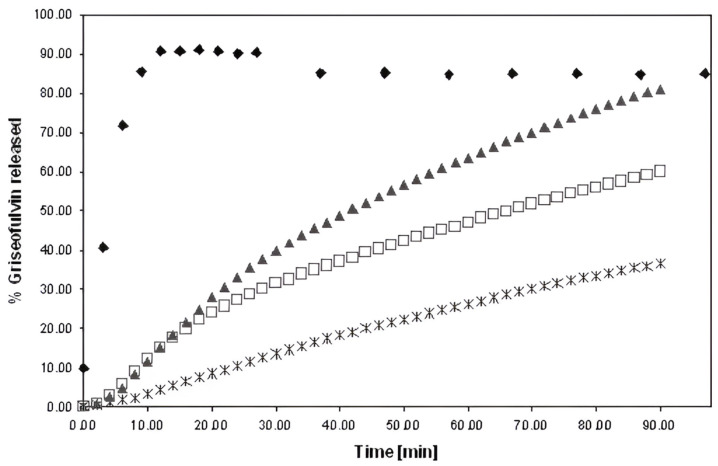
Dissolution profile of Griseofulvin in artificial gut fluid of pH 7.4; X shape shows Crystalline Griseofulvin, square shows Milled Griseofulvin, triangle shows RESS Griseofulvin, and Diamond shows aerogel Griseofulvin [150].

In a pharmaceutical aspect, aerogels have gained interest in the last decade due to their properties. For oral drug administration, several aerogel compositions and drugs were tested for release conditions and outcomes. For instance, ibuprofen impregnated into hollow silica aerogel microspheres (HSAMs) made from rice husk ash showed release properties significantly superior to the drug in its crystalline form [148]. Around 80% of the drug loaded into the gel was released in the first 15 min, in comparison to 11% from the crystalline drug, when placed in a solution of 0.1 N HCl with 1% sodium dodecyl sulphate solution. Overall, this study highlighted the capability of biocompatible and affordable rice husk-ash HSAMs to carry water-insoluble drugs. In another article, hybrid aerogels composed of silica and gelatine were designed to load hydrophobic acidic drugs (ibuprofen, ketoprofen, or triflusal) to help develop immediate- and sustained-release formulations [149]. These gels showed rapid release rates at an intestinal pH of 6.7 and sustained release rates at an acidic gastric pH of 2. In 2005, Smirnova et al. looked at the dissolution profile of four different Griseofulvin formulations: Crystalline Griseofulvin, Milled Crystalline Griseofulvin, RESS Crystalline Griseofulvin, and Griseofulvin aerogels [150]. The results showed aerogel Griseofulvin with a dissolution rate much higher than that of the nano micronized Griseofulvin (Figure 8). It was then concluded that to achieve rapid drug delivery, poorly soluble drugs may benefit more from aerogel delivery than micronization.

Several other in vivo and in vitro studies looking for poorly soluble molecules showed corresponding results when it comes to drug release rates and bioavailability from aerogel formulations [124,151,152,153,154].

In China, researchers from Tsinghua University documented their findings when inventing novel nanoscale capecitabine-loaded granules using silicon dioxide aerogels as a base [155]. They summarized that this method of drug delivery may provide higher oral bioavailability and lower toxicity in comparison to current oral formulations. It was further noted that, in comparison to existing liposome nanogranules and polymer nanoparticles, the production of these aerogel granules is simpler and more affordable [155].

For pulmonary administration, the use of aerogels to improve pharmacotherapeutic drug delivery was reported by Lee and Gould [129]. They documented that the porosity and surface area of aerogels is larger than that of spray-dried particles and that their density is lower than that of crystalline powders. This makes these drug-loaded aerogel particles exceptional for administration via inhalation, as they can float longer in the lungs, allowing for deeper delivery. Furthermore, these nanoporous particles have the capability to readily dissolve in pulmonary surfactants, creating an advantage [129]. The use of supercritical fluid technology to prepare aerogel microparticles has also been investigated for pulmonary drug-delivery formulations [156]. In this study, Salbutamol-loaded chitosan aerogel microparticles were developed and tested. Their sustained-release properties were then documented.

### 3.8. Aerogels for Antimicrobial Use

The processing and utilization of aerogels with antimicrobial properties has been documented well over the years. These antimicrobial gels are often developed using the natural antimicrobials, such as chitosan, as a base or by loading the gels with antibiotics or even metals such as silver. For instance, cellulose–chitosan aerogel microfibers have been studied for their use in wound dressing [157]. The processed fibers were loaded with ibuprofen to reduce pain, and they exhibited high moisture and water uptake, bactericidal properties, high porosity, and specific surface area, as well as biocompatibility [157]. Uddin et al. looked at the antimicrobial activity of cellulose nanofibrils (CNFs) which were loaded with either Lysozyme or silver nanoparticles (AgNPs) [158]. The gels were then tested against *Escherichia coli* and *Staphylococcus aureus*, where they were successfully able to inhibit bacterial growth.

López-Iglesias et al. developed vancomycin-loaded chitosan aerogel beads for chronic-wound healing [114]. It was determined that these loaded gels could provide rapid local administration of vancomycin at a debride wound site to prevent infections; thus, they have potential to be used in chronic-wound dressings. One recent study by Sanchez et al. looked at using CNF-lignin bioaerogels that contained iron for biomedical applications [159]. The properties of these gels were tested, and they exhibited strong antioxidant properties against reactive oxygen species. When loaded with tetracycline, these gels possessed potent antimicrobial activity. Their biocompatibility and sustained release properties were also highlighted, rendering the authors to conclude the promising use of these gels in wound dressings [159].

### 3.9. Summary of Aerogels Findings

1. More research is focusing on using aerogels for drug delivery.
2. The formulation of aerogel typically requires three major steps, which are sol–gel formation, aging, and finally drying.
3. Several strategies are utilized to load drugs into aerogels based on their solubility and stability.
4. Strategies of drug loading into aerogels include loading the drug into precursor solution, incorporating the drug into the gel after it is formed, introducing the drug into the gel during the supercritical drying stage, and adsorption precipitation.
5. Drug release from aerogels involves two mechanisms, which are drug dissolution and drug transport from the matrix to the dissolution media.
6. The mechanisms of drug release are influenced by physiochemical properties like hydration of the drug and matrix, among other factors.
7. The use of aerogels in drug delivery has several benefits, including versatile dosage forms, high porosity, low density, and rapid drug dissolution.
8. Aerogels still present with some limitations, including uncontrolled drug release, fragility, moisture sensitivity, and shrinkage at high temperatures.
9. Aerogels have several applications in various fields, including the environmental field, biomedicine, and pharmaceutical sciences.
10. Development of antimicrobial aerogels has several techniques, including using natural substances like chitosan or by incorporating antibiotics or metals such as silver.
11. Antimicrobial aerogels have prospective use in chronic-wound dressings and biomedical purposes, as they effectively suppress bacterial growth.

## 4. Comparison

### 4.1. Advantages and Disadvantages of Hydrogels and Aerogels


**Hydrogels**

**Aerogels**
**Advantages** [12,160,161,162]
Possess high degree of elasticity, making them comparable to tissues in natureBiocompatible and biodegradableUsed locally so that first-pass metabolism is passedLong-lasting and sustained activity compared to conventional drug-delivery methodsLower administration doseImprovements in drug use and patient complianceDefense against irritant medicines for mucosaCost-effective, since patient requires fewer dose units throughout therapy

Maintenance of drug levels for a prolonged time is required for drugs with narrow therapeutic index and for chronic treatmentsReducing the administration frequency could be achieved by prolonging the residence time of the delivery system in the gastrointestinal tractCan be used as solubilizing aidsThe high porosity and specific surface make aerogels appealing for pulmonary, nasal, or transdermal
**Disadvantages** [160,161,162,163]
They may cause a sensation felt akin to the movement of maggotsIn case of contact lenses, they cause eye irritationIn case of contact lenses, they may cause lens disposition if applied to the eyesThey are non-adherent and may need to be secured by a secondary dressing

High costProduction process is lengthy and exhaustingInorganic aerogels have many disadvantages, such as chemical toxicity, non-biodegradability, and low mechanical strength, that limits their usage in many health fields


### 4.2. Applications and Current Research and Development Trends of Aerogels and Hydrogels

To begin with, Aerogels have a wide range of applications in numerous areas because of their high porosity and nanoscale pore diameters (pore diameter < 50 nm). They have non-pharmaceutical applications, such as for thermal insulation in the construction/building [104] and aerospace domains, catalysis, environmental cleanup, chemical sensors, acoustic transducers, and energy-storage devices [164]. However, more recently, they are utilized as biomaterials for several medical and pharmaceutical applications due to multiple advantages. For example, aerogels have special properties, including large porosity, a controlled pore diameter, a large internal surface, and an interconnected 3D structure, together with biodegradability and proven biocompatibility [165]. Different aerogels have attracted tremendous interest of the biotechnology community for various potential applications, including disease diagnosis [147], ultrasound contrast agents, biomedical implantable devices, antibacterial materials, and drug delivery [16,96]. In particular, aerogels can be used for drug delivery, as the loading of the drug in the aerogels can be carried out either during the sol–gel process itself, during the solvent exchange of the gels, or by supercritical impregnation of aerogel, depending on the drug stability and affinity for solvents (water, organic solvent, and scCO_2_) and for the aerogel matrix [96]. The use of aerogels for drug-delivery systems was first evaluated using silica aerogels due to their biocompatibility and the broad knowledge of their sol–gel chemistry [115]. Drug-loaded silica aerogel can provide immediate or sustained release of the drug depending on the hydrophilic or hydrophobic nature of the obtained silica aerogel surface. Drug-loaded silica particles can be coated with polymeric coatings, conferring the system with the pH-controlled release of the drug specific to gastric or enteric delivery [166,167].

However, recently, bio-based aerogels are being actively studied as drug carriers since they combine their availability and renewability with the biodegradability that silica aerogels lack. The use of bio-based aerogels for drug delivery purposes has been reported from different sources like polysaccharides (starch, alginate, pectin, chitosan, and cellulose), proteins, or hybrids (gelatin–silica, cellulose–polyethyleneimine, and chitosan–silica) [168,169]. Bio-based aerogels have been used in the form of microspheres and have been loaded with medications such as nifedipine, benzoic acid, theophylline, ibuprofen, nicotinic acid, and ketoprofen via supercritical fluid-assisted impregnation [169].

One of the most important applications of aerogels is wound healing. Polysaccharide aerogels are able to uptake large amounts of exudate due to their nanoporous texture. During the exudate incorporation, the aerogel turns into a soft hydrogel that is able to swell and completely fill the wound site, preventing it from the formation of fluid-filled pockets that would enable bacterial proliferation [170]. In particular, aerogel particles based on polysaccharides (e.g., alginate and chitosan) are known as healing stimulators because they induce human cytokine production and macrophage activation. Thus, they are useful in improving the healing process, in addition to improving the release of the encapsulated drug into the wound site [95].

Besides the topical applications of aerogels in drug delivery, drug-containing aerogel powders of 0.5–10 μm for inhalation via the jet milling of aerogels containing the drug (insulin, morphine, and sildenafil citrate) were being heavily studied recently to achieve faster drug-delivery rates [171].

For hydrogels, recent studies reveal the wide range of their applications, including medication delivery, tissue engineering, regenerative medicine, agriculture, biomaterials, and the food sector. In particular, current research is focusing on smart hydrogels due to the ability of a smart hydrogel to change its properties (such as mechanical properties, swelling capacity, hydrophilicity, or permeability of bioactive molecules) under the effect of its surroundings, including temperature, pH, electromagnetic radiation, magnetic field, and biological factors [23]. There are numerous examples of applications of smart hydrogels in drug delivery. Firstly, dexamethasone hydrogel is a thermoresponsive hydrogel that can be used in osteoarthritis and rheumatoid arthritis. Under temperature changes, the matrix volume of thermo-responsive hydrogels (TRHs) can be changed due to expansion or contraction, and the solubility, conformation, and phase transition of the polymer may change. HPMA-copolymer-based dexamethasone can change its shape from a liquid form at 4 °C to a hydrogel form at 30 °C or higher by increasing the concentration of Dex. This HPMA–Dex can become a hydrogel (ProGel-Dex) after intra-articular administration in rodent models of inflammatory arthritis and osteoarthritis [172]. Moreover, bortezomib is one example of a pH-responsive hydrogel, and it can be used for colorectal cancer. pH-responsive hydrogels can swell depending on the change in the pH value in the surrounding environment. During the swelling period, the interior structure of pH-responsive hydrogels contains the absorbed water, resulting in the embedded drugs being released. This hydrogel can be used to release the drug in the stomach or intestine through oral administration. Tumor environments are usually acidic; thus, pH-responsive hydrogels can be a drug carrier for anticancer therapy. Bortezomib (BTZ), a new chemotherapy drug for colorectal cancer, is conjugated with LUL (luteolin) in mPEG-LUL by a borate ester bond to form mPEG-LUL-BZT. This hydrogel can sustain and release BTZ at pH 5.5 for up to 50 h [173].

Besides those applications, hydrogels are commonly used in wound dressing. Several studies have shown that hydrogels can form a physical barrier and remove excess exudate. They also provide a moist environment to promote the process of wound healing. In addition, hydrogels can be applied in sprayable- or injectable-formulation wound dressing, which may fill irregularly shaped wounds [174,175,176]. Recently, functional hydrogels which are developed by using different polymers and bioactive factors, have received a lot of attention in wound-dressing research. These hydrogels exhibit high-performance biological activities, such as anti-infective properties and promoting blood coagulation [177]. Therefore, one area to look forward to in the future will be how to modify and enhance the capabilities of those functional hydrogels to maximize their benefit in different pharmaceutical applications.

## 5. Discussion and Overall Summary of Findings

The formulation of hydrogels is a meticulous process that begins with the careful selection of polymers, cross-linking agents, and additives tailored to specific pharmaceutical applications. Among the critical initial steps is the choice of polymer, wherein hydrophilic options like polyacrylic acid, polyvinyl alcohol, and polyethylene glycol are preferred for their exceptional ability to absorb and retain water while maintaining structural integrity [19]. The subsequent cross-linking process is essential for stabilizing the polymer chains, forming a stable 3D network crucial for preventing hydrogel dissolution before use.

There are two main types of hydrogels based on the cross-linking process: physically cross-linked hydrogels and chemically cross-linked hydrogels. Physically cross-linked hydrogels rely on non-covalent interactions, exhibiting fragility and reversible responses to external stimuli, such as changes in temperature and pH [19]. Various methods, including ionic cross-linking, stereo-complex formation, and hydrophobic modification of polysaccharides, contribute to the diverse array of physically cross-linked hydrogels. On the other hand, chemically cross-linked hydrogels involve covalent bonding, resulting in permanent structures that do not dissolve in the surrounding medium and do not exhibit reversible responses to environmental changes. Common chemical cross-linking methods include chain-growth polymerization, gamma and electron beam polymerization, and addition/condensation polymerization [20,21].

Despite the advantages of hydrogels in drug delivery, such as controlled release and biocompatibility, they do present limitations. These include low mechanical strength, restricted drug loading for hydrophobic drugs, and challenges in application methods, especially for injectables. The need for careful consideration of these factors is crucial in ensuring the reliability and practicality of hydrogel-based drug-delivery systems [21].

Hydrogels exhibit unique physical characteristics that position them as versatile carriers for controlled drug release [21], with applications spanning ocular, oral, transdermal, and injectable drug-delivery systems [8]. In ocular drug delivery, hydrogels like Restasis provide sustained release, improving bioavailability and reducing the need for frequent administration [23]. In oral drug delivery, hydrogels protect drugs from the harsh conditions in the stomach, enabling controlled release in the intestines [22]. Transdermal drug delivery, exemplified by the fentanyl patch, relies on hydrogels for sustained drug release, ensuring constant concentration in the systemic circulation [22,25]. Injectable drug-delivery systems, such as Lupron Depot, utilize hydrogels for localized and controlled drug delivery, eliminating the need for frequent injections. The application of hydrogel-based drug-delivery systems, such as diclofenac sodium gel, demonstrates advantages in terms of non-invasiveness and improved absorption rates compared to other formulations [21,22,26].

However, the limitations of hydrogels, such as low mechanical strength, rapid drug release, and potential toxicity from chemical cross-linking agents, need to be carefully considered for practical use in clinical settings. Despite these challenges, ongoing research is focused on overcoming these limitations and enhancing the efficacy of hydrogel-based drug-delivery systems [8,21,34,35].

Furthermore, hydrogels play a significant role in antimicrobial applications, particularly in wound healing. Recent studies highlight the development of hydrogel patches loaded with antimicrobial agents like neomycin to enhance wound healing. These hydrogel formulations offer controlled delivery, efficient spreadability, and reduced irritation, presenting promising options for addressing bacterial skin and soft tissue infections. The evaluation of hydrogel-based formulations, such as those containing cefotaxime, showcases their potential in providing sustained antibacterial activity, offering advantages in terms of spreadability, drug release, and in vivo performance [30,31].

Overall, hydrogels represent a versatile and promising platform for drug-delivery applications, providing controlled release, biocompatibility, and potential solutions to various pharmaceutical challenges. However, their limitations, including mechanical strength, drug-loading capacity, and application challenges, should be carefully considered. Advances in hydrogel formulations, especially in antimicrobial applications, demonstrate ongoing efforts to overcome these limitations and enhance the efficacy of hydrogel-based drug-delivery systems.

Moving on to aerogels, their production involves a multistep process encompassing sol–gel formation, aging, and drying [14,34,40]. This intricate process begins by converting gel precursors into hydrogels or alcogels through chemical cross-linkers or alterations in temperature or pH. The subsequent steps involve aging the gels in a water and alcohol solution, followed by washing or solvent exchange to remove the initial solvent. The drying stage then eliminates residual solvent from the pores while preserving the outer network structure [16,34,35].

Aerogels exhibit versatility in formulations, ranging from beads and spheres to powders, sheets, and coatings, allowing adaptation to diverse applications [14,86,107]. In the context of drug delivery, the preference for spherical aerogels is emphasized for enhanced flowability, reproducibility, ease of handling, increased surface area, and reduced risk of inflammatory responses [14,34].

The loading of drugs into aerogels employs various strategies based on solubility and stability. Methods include incorporating drugs into the precursor solution, soaking the gel in a drug-containing solution after formation, introducing drugs during supercritical drying, or impregnating drugs into pre-formed aerogels through scCO_2_. Each method presents distinct advantages and challenges, influencing factors like drug solubility, diffusion rates, and homogeneity of drug distribution [12,16,37,40,43,53].

Drug release from aerogels involves mechanisms of dissolution and transport, influenced by physiochemical properties, hydration, and interactions between the drug and the gel network. Hydrophilicity and hydrophobicity play crucial roles in the dissolution rate, impacting drug-delivery kinetics [12,110].

Aerogels offer several advantages in drug delivery, including the ability to formulate diverse dosage forms, high porosity facilitating liquid absorption, and rapid drug dissolution for quicker absorption. Their low density is advantageous in pulmonary drug administration, while the large surface area contributes to improved drug dissolution rates. However, limitations exist, such as potential uncontrolled drug release due to the broad surface area, brittleness of silica aerogels, and moisture sensitivity [12,65,66,67,68,69,70].

Despite these drawbacks, aerogels find applications in various fields, including aeronautics, environmental cleanup, catalysts, and biomedical uses. Biocompatibility studies have shown promising results, and aerogels have demonstrated potential in wound healing, oral drug administration, and pulmonary drug delivery. Antimicrobial aerogels, incorporating natural antimicrobials or loading with antibiotics or metals, show promise in applications such as wound dressing and chronic wound healing, emphasizing their versatility and potential in biomedical contexts [34,53,54,55,56,57,72,73,80,81,82,83,111,112,113].

The advantages and disadvantages of hydrogels and aerogels present a promising route for drug delivery and biomedical applications. Hydrogels, characterized by their high elasticity resembling natural tissues, biocompatibility, and biodegradability, offer benefits such as sustained drug activity, lower administration doses, and improved patient compliance. They are particularly effective in defending against irritant medicines for mucosa. However, challenges arise in maintaining drug levels for an extended period, necessitating prolonged residence times in the gastrointestinal tract for drugs with narrow therapeutic indices or for chronic treatments. The non-adherent nature of hydrogels may require additional securing measures.

On the other hand, aerogels boast high porosity and a specific surface, making them attractive for various applications, including thermal insulation, catalysis, environmental cleanup, and drug delivery. Their unique properties, such as controlled pore diameter and biodegradability, position them as versatile biomaterials. Bio-based aerogels, derived from polysaccharides and proteins, have gained attention for drug delivery due to their renewability and biodegradability. Applications of aerogels in wound healing, disease diagnosis, ultrasound contrast agents, and biomedical implantable devices showcase their diverse potential.

While hydrogels find applications in medication delivery, tissue engineering, and wound dressing, the focus is shifting toward smart hydrogels with responsive properties influenced by environmental factors. This paper highlighted the ongoing research trends, diverse applications, and future prospects for both hydrogels and aerogels in the field of pharmaceuticals and biomaterials. It underscores the need for a comprehensive understanding of their advantages and limitations to harness their full potential in advancing drug delivery and biomedical applications.

## 6. Conclusions

In conclusion, research into gels—especially hydrogels and aerogels—has revealed a wide range of substances with special qualities and uses. Hydrogels present a promising path for a wide range of applications, from environmental to biomedical, due to their unique formulation and water-absorbing properties. Nevertheless, their drawbacks—need for secondary dressing and possible toxicity, for example—highlight the necessity of ongoing research to overcome these obstacles. Conversely, aerogels, which are renowned for their exceptionally light weight and porous architectures, show great promise in many areas, such as medicine delivery and insulation. Their benefits, such as their porosity and long-lasting properties, make them of great scientific interest despite the high cost and the complex processes required in their creation.

This comparative review studied the mechanical properties, uses, and formulation procedures of hydrogels and aerogels, revealing clear benefits and drawbacks. Looking ahead, the application-focused emphasis on antimicrobial use in hydrogels and aerogels signals the growing importance of these formulations in tackling modern challenges like infection management. The combination of innovative formulas and smart design in hydrogels and aerogels creates new opportunities for ground-breaking medical and environmental solutions, among other applications.

To fully utilize the benefits of hydrogels and aerogels and get over their limitations, ongoing research and development efforts are crucial for transformative advancements in diverse fields.

## Figures and Tables

**Table 2:** Application of in situ gelling hydrogels in drug delivery.

Hydrogels	Drug	Materials	Sustained-Release Time	Proposed Applications	Reference
Thermoresponsive hydrogel	Topotecan	Poloxamer 407 and poloxamer 188	28 days	Colorectal cancer	[41]
	Dexamethasone	HPMA	>30 days	Osteoarthritis and rheumatoid arthritis	[42]
	Lamivudine and zidovudine	Pluronic F-127	168 h	AIDS	[43]
pH-responsive hydrogel	Bortezomib	MPEG-LUT	50 h	Colorectal cancer	[44]
Photoresponsive hydrogel	Doxycycline	SPCOOH-modified silicone hydrogel (poly(HEMA-co-PEGMEA)	42 h	Inflammation	[45]
Dual-responsive hydrogel (pH/thermo)	Methotrexate		50 h	Breast cancer	[46]
	Doxorubicin chemosensitizer curcumin	Poly (NIPAAm-co-DMAEMA)	168 h	Colon cancer	[47]

**Table 4:** Applications of hydrogels for 3D cell culture.

Source of Hydrogels	Properties	Materials	Cell	Applications
Natural	Provides comparable viscoelasticity and fibrils to the ECM; having good biocompatibility; endogenous factors can support cellular activity.	Alginate	hESCs/hiPSCs [72], hiPSCs-derived neurons [73]	Enhance the generation of retinal pigmented epithelium and neural retina of hESCs/hiPSCs; form complex neural networks.
Natural		Collagen	Rat chondrocyte [74], hMSCs, rMSC, HUVECs/hASCs.	Maintain the chondrocyte phenotype; facilitate chondrogenic differentiation of hBMSCs [75] and rBMSCs; form stable EC networks; promote cell viability; promote growth of hMSCs [76].
Natural		Fibrin	HUVECs/hMSCs [77], porcine cumulus–oocyte complexes (COCs), primary human chondrocytes, mHPSCs, and hiPSCs/HUVECs/human dermal fibroblast.	Prevascular formation of HUVECs, improve cell proliferation of hMSCs and enhance their osteogenic differentiation and bone mineral deposition; maintain the functional relationship between oocytes and follicular cells [78]; induce the production of glycosaminoglycans and collagen type II of primary human chondrocytes [79].
Synthetic	Have a good mechanical strength to provide structural support for various cell types in 3D cell culture.	PEG	HiPSCs, mMSCs, chondrocyte, and hMSCs (human mesenchymal stem cells).	Enhance the hematopoietic differentiation of hiPSCs [80]; evaluate the behavior of mMSCs and hMSCs at the specific condition; prolong the oxygen release of chondrocytes [81].
		PVA	MHSCs, mSCCs, human glioma cell lines LN299, U87MG and Gli36, human breast cancer Hs578T cells, and human pancreatic cancer cell lines Sui67 and Sui72.	Enhance the expansion of murine hematopoietic stem cells (mHSCs) [82]; promote the meiotic and post-meiotic differentiation rate of mSCCs [83]; form tumor spheroids.

**Table 5:** Controlled nonviral gene delivery from natural- and synthetic-based hydrogels.

Type of Polymer	Polymer	System	NA type	Study	Application	Reference
Natural	Pullulan	Cationized pullulan hydrogel	SiRNA against MMP-2 (DEAE-pullulan complexes)	In vivo (implantation in rabbits)	Cardiovascular tissue repair	[85]
Natural	Collagen	PCLEEP nanofibers–collagen hydrogel	miRNA-222 (PCL-PPEEA micellar NPs)	In vivo (rat spinal cord incision model)	Nerve repair	[86]
Natural	Alginate	Alginate hydrogel	pDNA encoding for BMP-2	In vitro (MSCs)/In vivo (s.c. dorsal pocket from nude mice)	Bone repair	[87]
Synthetic	Polyurethane	Polyurethane hydrogel	pDNA encoding for GATA4 (naked, microextrusion-based transfection system)	In vitro (hUC-MSCs)	Cardiovascular tissue repair	[88]
Synthetic	Poly(organophosphazene)	Poly(organophosphazene) thermosensitive hydrogel	pDNA (GC-g-PEI complexes)	In vitro (HepG2 cells)/In vivo (injection in mice)	Hepatocyte targeting	[89]
Synthetic	PNIPAm	PNIPAm/LDH hydrogel	siRNA against GAPDH (LPF lipoplexes)	In vivo (s.c. injection in mice)	Cartilage repair	[90]

## Data Availability

Not applicable.
